# Supplementary material for: The Ube2m-Rbx1 neddylation-Cullin-RING-Ligase proteins are essential for the maintenance of Regulatory T cell fitness
Source: Nat Commun. 2022 May 31;13:3021. doi: 10.1038/s41467-022-30707-8 (PMC9156764; doi:10.1038/s41467-022-30707-8)
Supplement: Supplementary file 1 — Supplementary Information [file 41467_2022_30707_MOESM1_ESM.pdf]

Supplementary Figures

The Ube2m-Rbx1 neddylation-Cullin-RING-Ligase proteins are essential for the maintenance of Regulatory T cell fitness

Di Wu[1,2,3], Haomin Li[4], Mingwei Liu[5], Jun Qin[5], Yi Sun[1,2,3*]

[1]Cancer Institute of the Second Affiliated Hospital and Institute of Translational Medicine, Zhejiang University School of Medicine, Hangzhou, Zhejiang, China, 310029.

[2]Cancer Center, Zhejiang University, Hangzhou, Zhejiang, China, 310058.

[3]Research Center for Life Science and Human Health, Binjiang Institute of Zhejiang University, Hangzhou, Zhejiang, China, 310053.

[4]Children's Hospital, Zhejiang University School of Medicine, Hangzhou, Zhejiang, China, 310003.

[5]State Key Laboratory of Proteomics, Beijing Proteome Research Center, National Center for Protein Sciences (Beijing) and Institute of Lifeomics, Beijing, China, 102206.

* To whom correspondence should be addressed: yisun@zju.edu.cn.

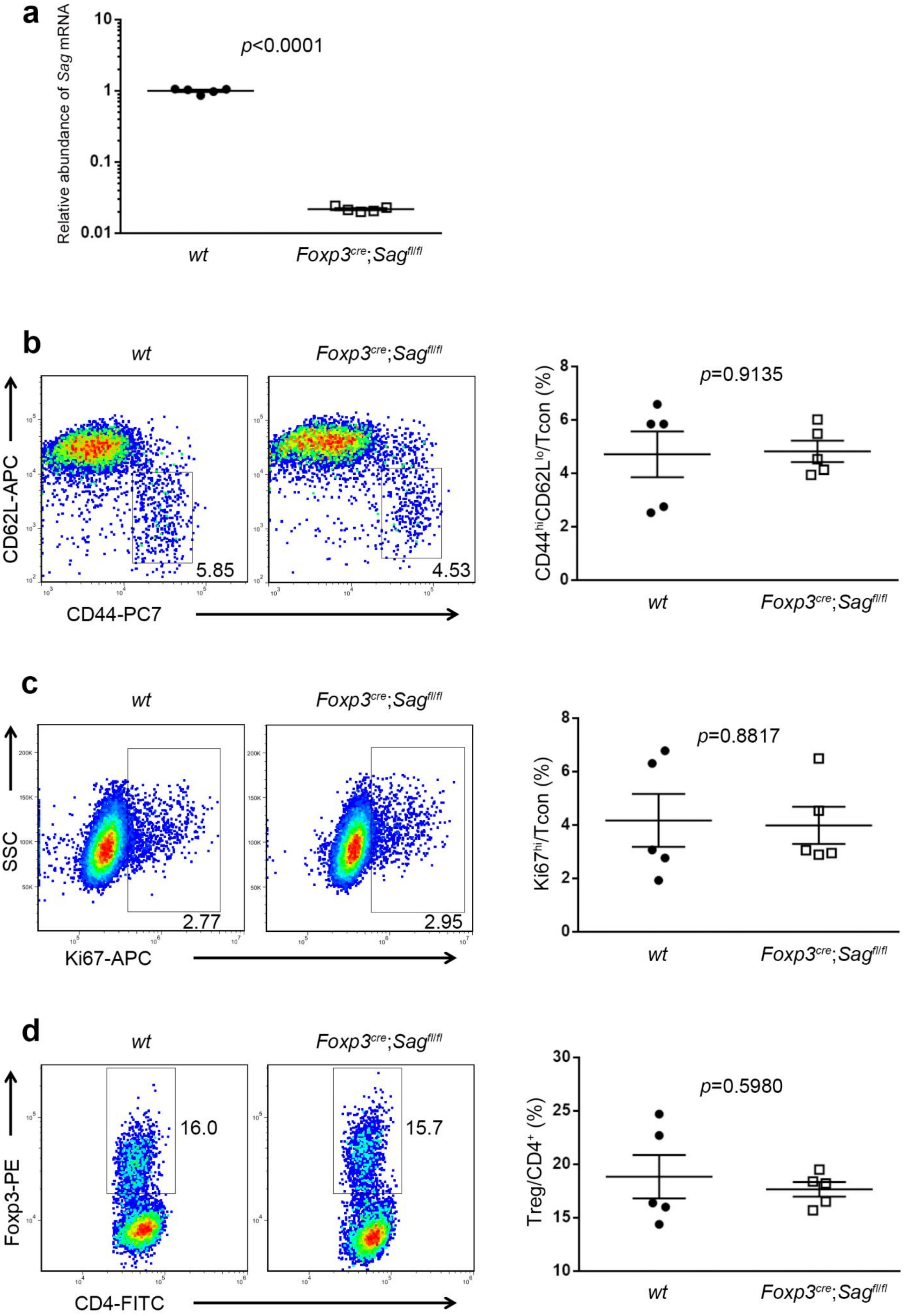

a
Relative abundance of Sag mRNA
10
1
0.1
0.01
p<0.0001
wt
Foxp3cre;Sagfl/fl
b
wt
Foxp3cre;Sagfl/fl
CD62L-APC
CD44-PC7
5.85
4.53
CD44hiCD62Llo/Tcon (%)
p=0.9135
c
SSC
Ki67-APC
2.77
2.95
Ki67hi/Tcon (%)
p=0.8817
d
Foxp3-PE
CD4-FITC
16.0
15.7
Treg/CD4+ (%)
p=0.5980

Supplementary Figure 1. Deficiency of *Rbx2*/*Sag* does not obviously impair Treg cell fitness at steady status

(a) Expression of *Sag* mRNA in $CD4^{+}YFP^{+}$ Treg cells from peripheral lymph nodes of *wt* and $Foxp3^{cre}$;$Sag^{fl/fl}$ mice, revealed by q-PCR (*n* =5 biologically independent samples from both male and female mice, $p<0.0001$).

(b) Expression of CD44 and CD62L in Tcon cells from peripheral lymph nodes of *wt* and $Foxp3^{cre}$;$Sag^{fl/fl}$ mice (10 weeks old, *n*=5 biologically independent samples from both male and female mice).

(c) Expression of Ki67 in Tcon cells from peripheral lymph nodes of *wt* and $Foxp3^{cre}$;$Sag^{fl/fl}$ mice (10 weeks old, *n*=5 biologically independent samples from both male and female mice).

(d) The proportion of Treg cells among $CD4^{+}$-T cells from peripheral lymph nodes of *wt* and $Foxp3^{cre}$;$Sag^{fl/fl}$ mice (10 weeks old, *n*=5 biologically independent samples from both male and female mice).

All error bars represent the SEM, data are presented as mean values +/- SEM. The *p* values were calculated by Mann–Whitney test. Source data are provided as a Source Data file.

a

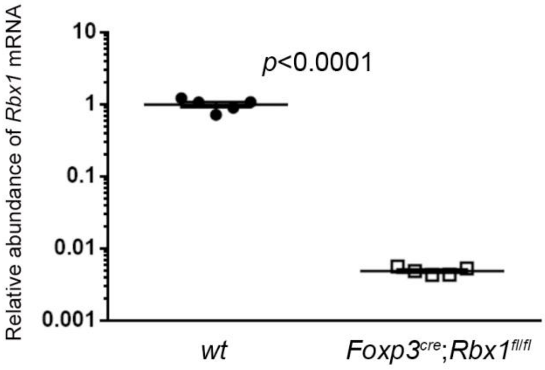

Relative abundance of Rbx1 mRNA
10
1
0.1
0.01
0.001
p<0.0001
wt
Foxp3cre;Rbx1fl/fl

b

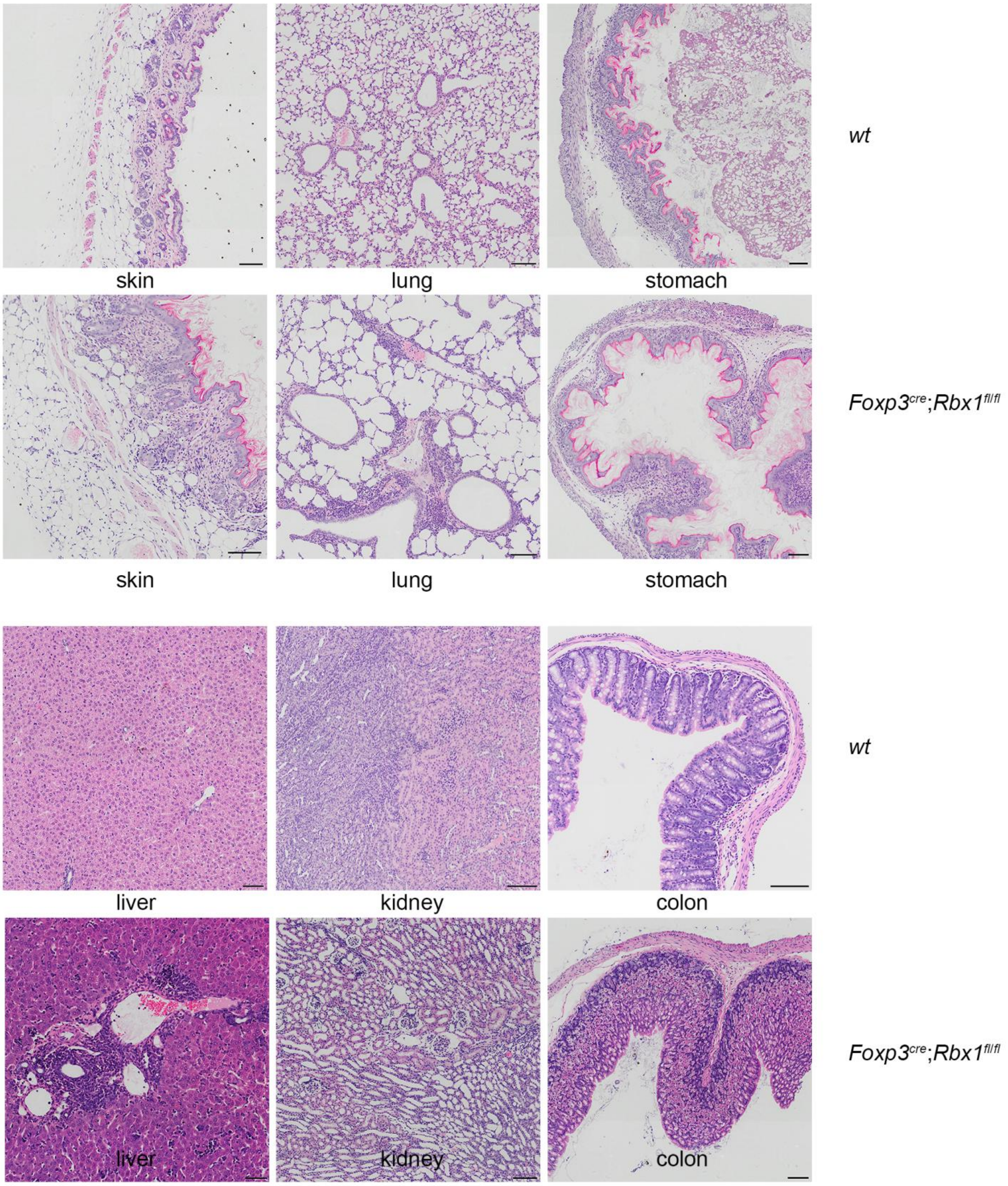

skin
lung
stomach
wt
skin
lung
stomach
Foxp3cre;Rbx1fl/fl
liver
kidney
colon
wt
liver
kidney
colon
Foxp3cre;Rbx1fl/fl

Supplementary Figure 2. Disastrous auto-immune disorders in *Foxp3*^{cre};*Rbx1*$^{fl/fl}$ mice

(a) Expression of *Rbx1* mRNA in CD4^{+}YFP^{+} Treg cells from peripheral lymph nodes of *wt* and *Foxp3*^{cre};*Rbx1*$^{fl/fl}$ mice. Left, revealed by transcriptome analysis; right, revealed by q-PCR (n =5 biologically independent samples from both male and female mice, p<0.0001). All error bars represent the SEM, data are presented as mean values +/- SEM. The p values were calculated by Mann–Whitney test. Source data are provided as a Source Data file.

(b) H&E staining of the skin, lung, stomach, liver, kidney, and colon from *wt* and *Foxp3*^{cre};*Rbx1*$^{fl/fl}$ mice (p19-20, scale bar = 50μm in liver, or 100μm in other organs, male, 3 times each experiment was repeated independently with similar results).

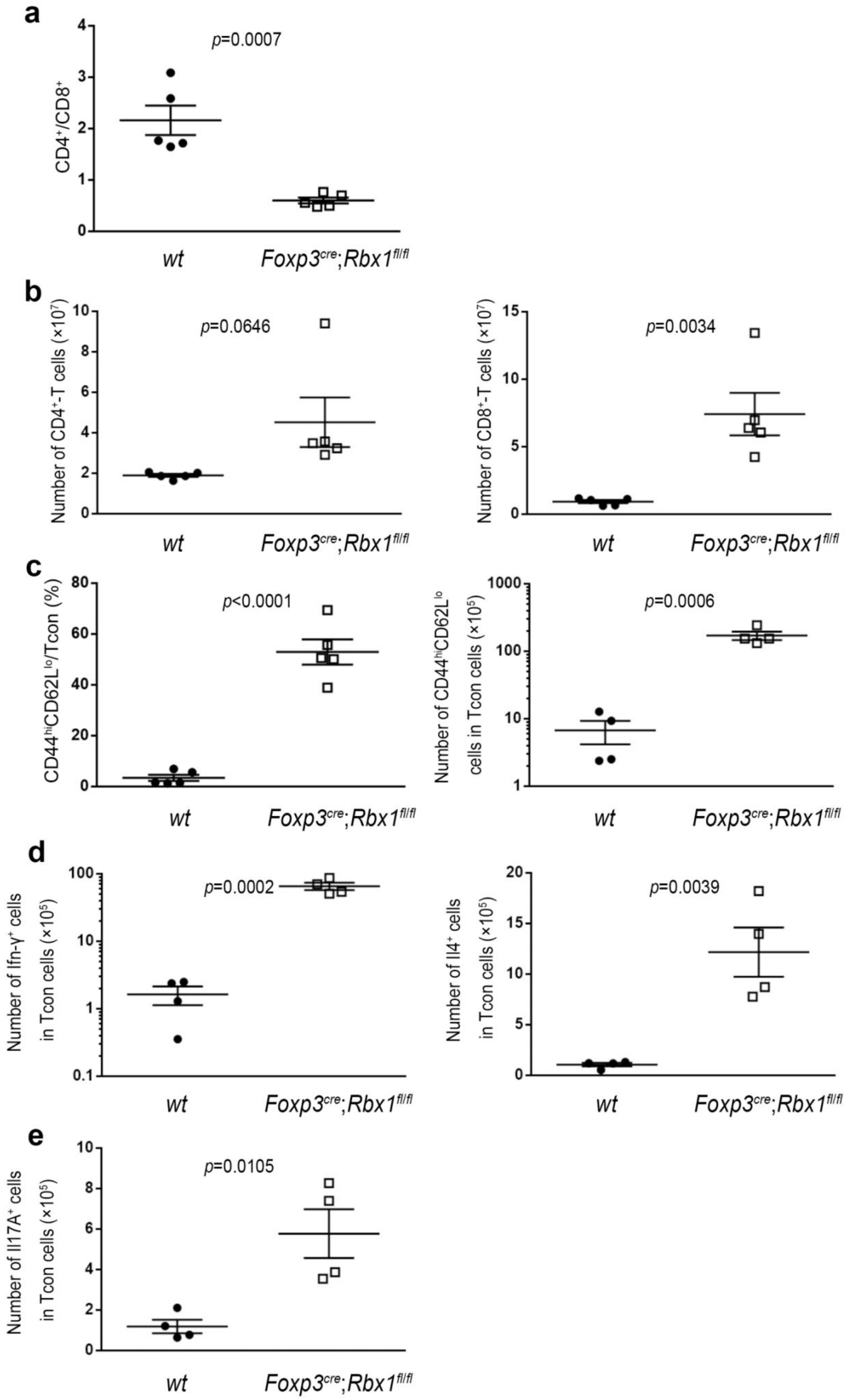

a
CD4+/CD8+
p=0.0007
0
1
2
3
4
wt
Foxp3cre;Rbx1fl/fl
b
Number of CD4+-T cells (×10⁷)
p=0.0646
0
2
4
6
8
10
wt
Foxp3cre;Rbx1fl/fl
Number of CD8+-T cells (×10⁷)
p=0.0034
0
5
10
15
wt
Foxp3cre;Rbx1fl/fl
c
CD44hiCD62Llo/Tcon (%)
p<0.0001
0
20
40
60
80
wt
Foxp3cre;Rbx1fl/fl
Number of CD44hiCD62Llo cells in Tcon cells (×10⁵)
p=0.0006
1
10
100
1000
wt
Foxp3cre;Rbx1fl/fl
d
Number of Ifn-γ+ cells in Tcon cells (×10⁵)
p=0.0002
0.1
1
10
100
wt
Foxp3cre;Rbx1fl/fl
Number of Il4+ cells in Tcon cells (×10⁵)
p=0.0039
0
5
10
15
20
wt
Foxp3cre;Rbx1fl/fl
e
Number of Il17A+ cells in Tcon cells (×10⁵)
p=0.0105
0
2
4
6
8
10
wt
Foxp3cre;Rbx1fl/fl

Supplementary Figure 3. Changes of ratios and numbers of immune cells in *Foxp3^{cre}*;*Rbx1$^{fl/fl}$* mice

(a) $CD4^+/CD8^+$ ratios in peripheral lymph nodes from *wt* and *Foxp3^{cre}*;*Rbx1$^{fl/fl}$* mice (p19-23, *n* =5 biologically independent samples from both male and female mice).

(b) Numbers of $CD4^+$ and $CD8^+$-T cells in peripheral lymph nodes from *wt* and *Foxp3^{cre}*;*Rbx1$^{fl/fl}$* mice (p19-23, *n*=5 biologically independent samples from both male and female mice).

(c) Ratio and Number of $CD44^{hi}CD62L^{lo}$ cells in Tcon cells from peripheral lymph nodes of *wt* and *Foxp3^{cre}*;*Rbx1$^{fl/fl}$* mice (p19-23, *n*=5 or 4 biologically independent samples respectively from both male and female mice, p<0.0001 (left) and p=0.0006 (right)).

(d) Number of Ifn-γ^+ and $Il4^+$ cells in Tcon cells from peripheral lymph nodes of *wt* and *Foxp3^{cre}*;*Rbx1$^{fl/fl}$* mice (p19-23, *n*=4 biologically independent samples from both male and female mice).

(e) Number of $Il17A^+$ cells in Tcon cells from peripheral lymph nodes of *wt* and *Foxp3^{cre}*;*Rbx1$^{fl/fl}$* mice (p19-23, *n*=4 biologically independent samples from both male and female mice).

All error bars represent the SEM, data are presented as mean values +/- SEM. The *p* values were calculated by Mann–Whitney test. Source data are provided as a Source Data file.

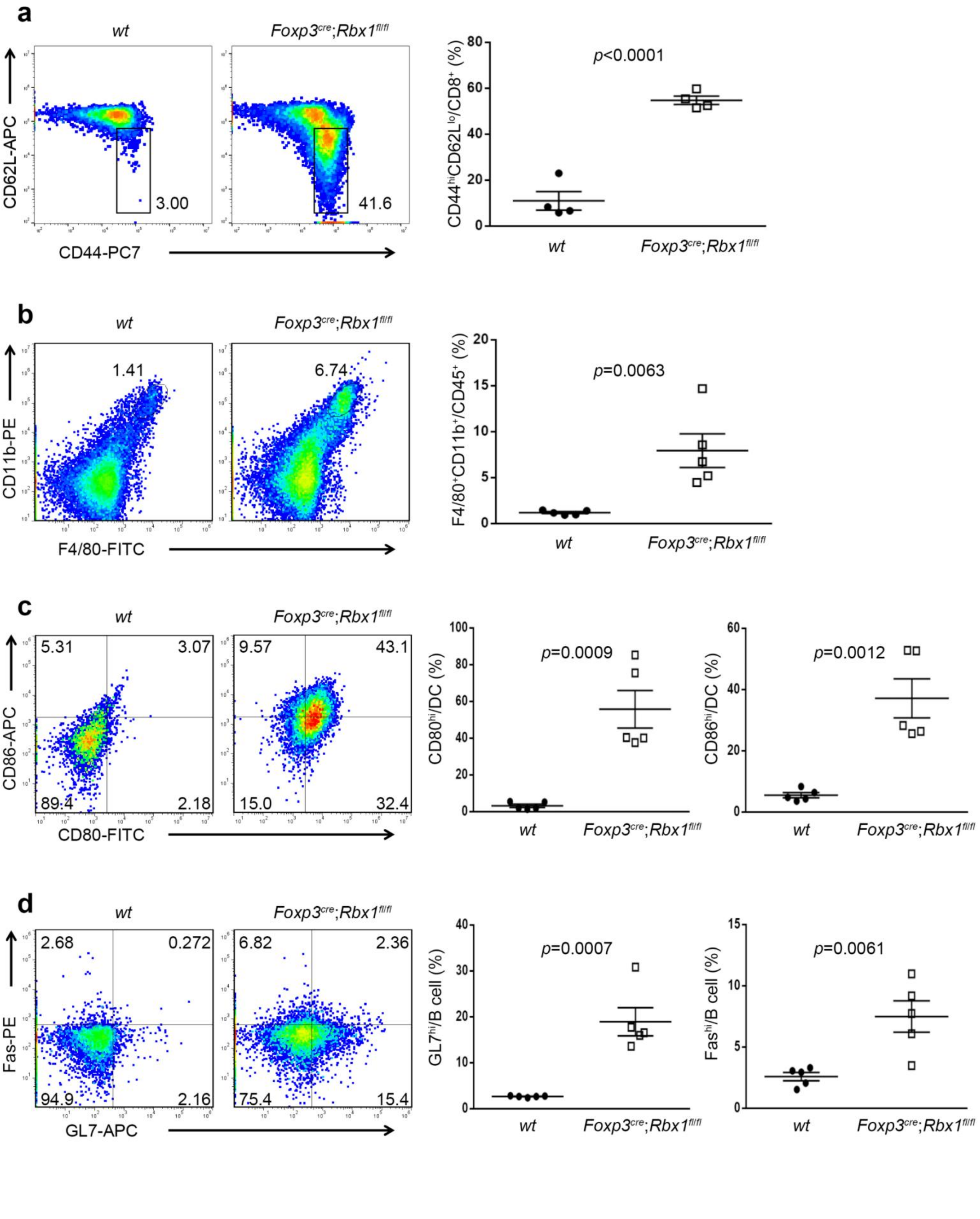

a
wt
Foxp3cre;Rbx1fl/fl
CD62L-APC
CD44-PC7
3.00
41.6
CD44hiCD62Llo/CD8+ (%)
p<0.0001
b
CD11b-PE
F4/80-FITC
1.41
6.74
F4/80+CD11b+/CD45+ (%)
p=0.0063
c
CD86-APC
CD80-FITC
5.31
3.07
89.4
2.18
9.57
43.1
15.0
32.4
CD80hi/DC (%)
p=0.0009
CD86hi/DC (%)
p=0.0012
d
Fas-PE
GL7-APC
2.68
0.272
94.9
2.16
6.82
2.36
75.4
15.4
GL7hi/B cell (%)
p=0.0007
Fashi/B cell (%)
p=0.0061

Supplementary Figure 4. Over-activation of multiple types of immune cells in *Foxp3^{cre};Rbx1$^{fl/fl}$* mice

(a) Expression of CD44 and CD62L in $CD8^+$-T cells from peripheral lymph nodes of *wt* and *Foxp3^{cre};Rbx1$^{fl/fl}$* mice (p19-23, n=4 biologically independent samples from both male and female mice, p<0.0001).

(b) Percentage of macrophages ($F4/80^+CD11b^+$) among $CD45^+$ cells from peripheral lymph nodes of *wt* and *Foxp3^{cre};Rbx1$^{fl/fl}$* mice (p19-21, n=5 biologically independent samples from both male and female mice).

(c) Expression of CD80 and CD86 in dendritic cells from peripheral lymph nodes of *wt* and *Foxp3^{cre};Rbx1$^{fl/fl}$* mice (p19-21, n=5 biologically independent samples from both male and female mice).

(d) Expression of GL7 and Fas in B cells from peripheral lymph nodes of *wt* and *Foxp3^{cre};Rbx1$^{fl/fl}$* mice (p19-21, n=5 biologically independent samples from both male and female mice).

All error bars represent the SEM, data are presented as mean values +/- SEM. The *p* values were calculated by Mann–Whitney test. Source data are provided as a Source Data file.

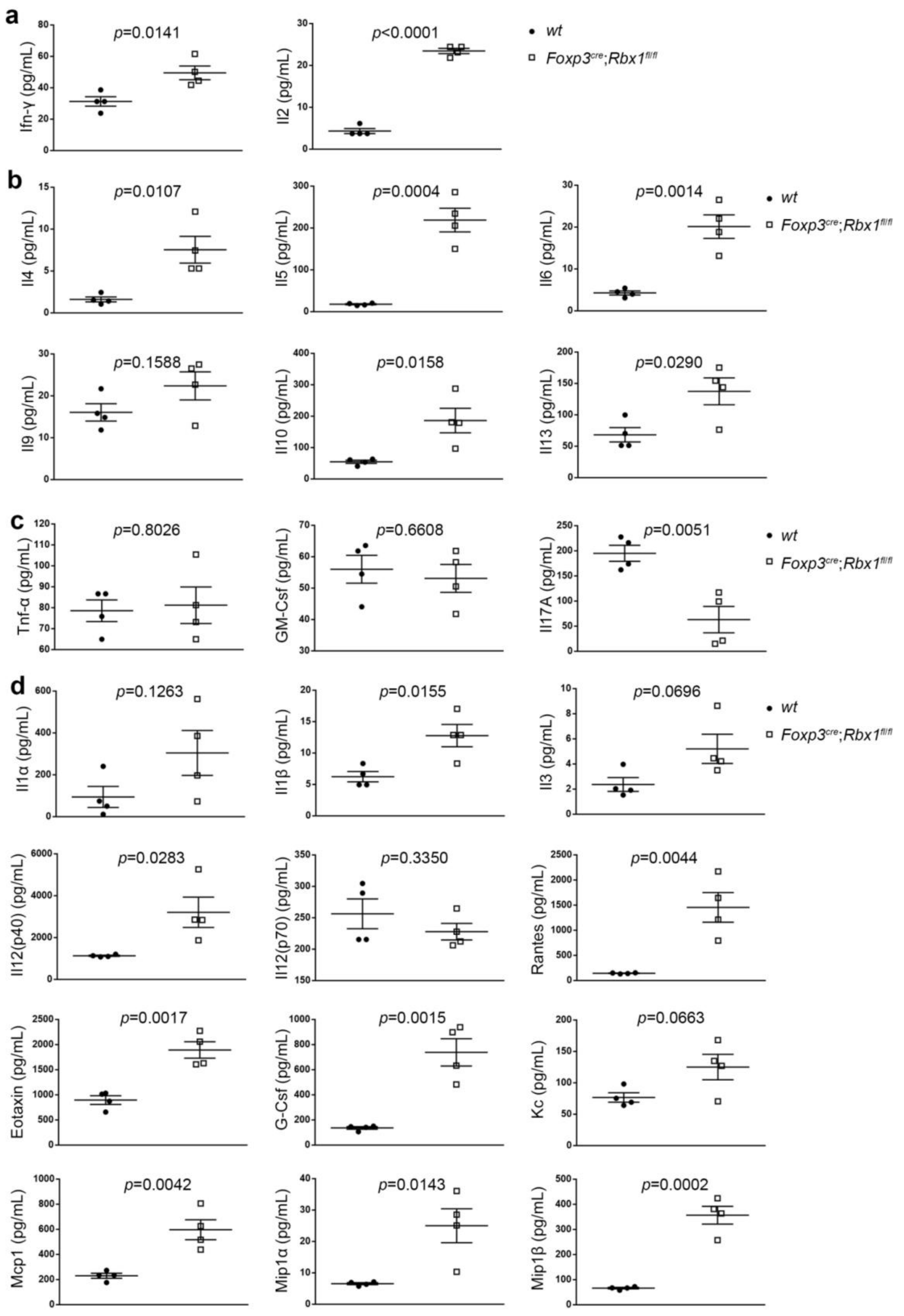

Supplementary Figure 5. Quantification of serum cytokines in *wt* and *Foxp3^{cre}*;*Rbx1$^{fl/fl}$* mice (p16-19, *n* =4 biologically independent samples from both male and female mice)

(a) T_H1 cytokines (*p*=0.0141 (left) and *p*<0.0001 (right)). **(b)** T_H2 cytokines. **(c)** T_H17 cytokines. **(d)** Other cytokines.

All error bars represent the SEM, data are presented as mean values +/- SEM. The *p* values were calculated by Mann–Whitney test. Source data are provided as a Source Data file.

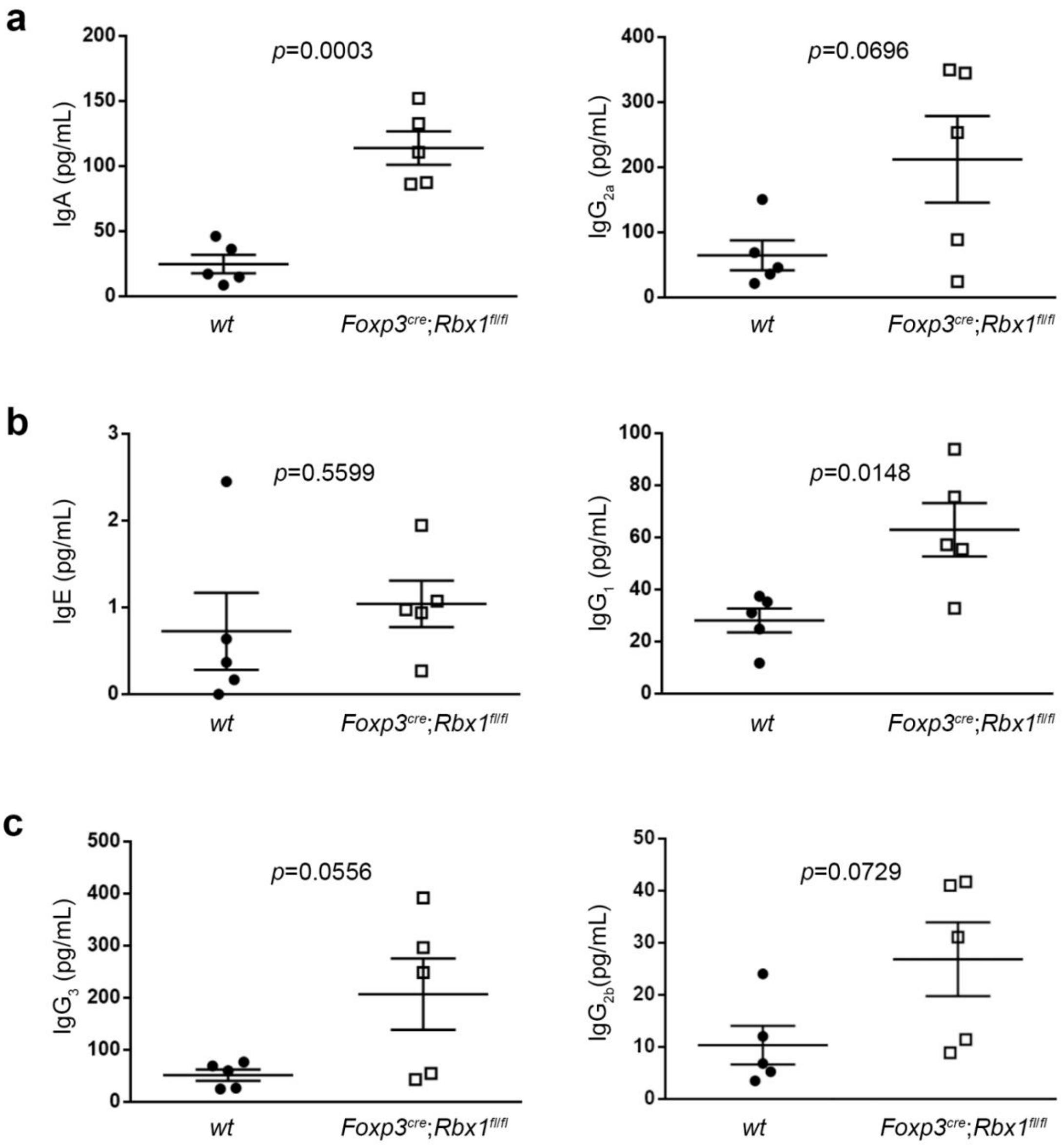

Supplementary Figure 6. Quantification of serum immunoglobulin subclasses in *wt* and *Foxp3^cre^;Rbx1^fl/fl^* mice (p20-21, *n* =5 biologically independent samples from both male and female mice)

(a) T_H1 antibodies.

(b) T_H2 antibodies.

(c) T_H17 antibodies.

All error bars represent the SEM, data are presented as mean values +/- SEM. The *p* values were calculated by Mann–Whitney test. Source data are provided as a Source Data file.

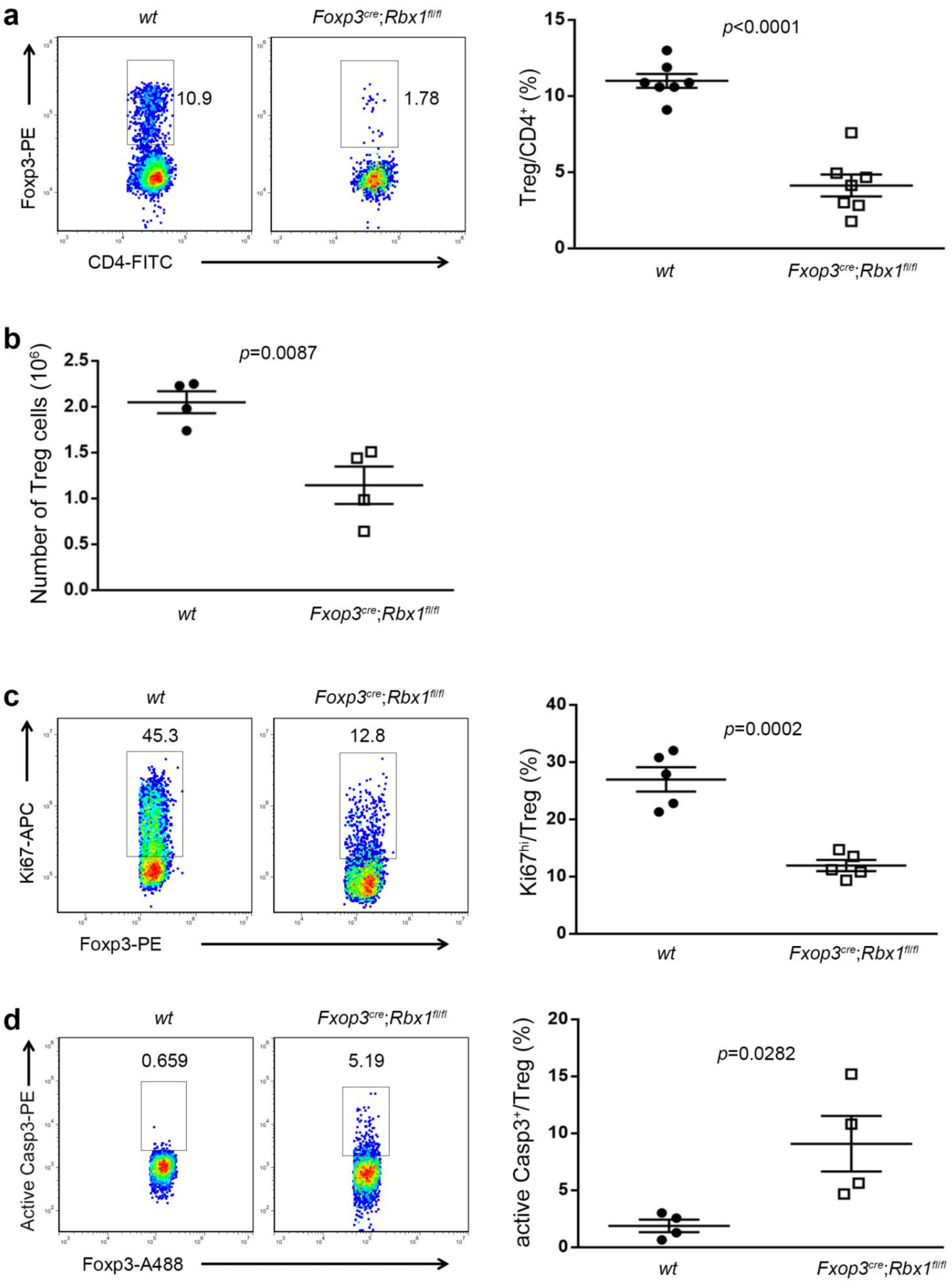

a
wt
Foxp3cre;Rbx1fl/fl
10.9
1.78
Foxp3-PE
CD4-FITC
p<0.0001
Treg/CD4+ (%)
wt
Fxop3cre;Rbx1fl/fl
b
p=0.0087
Number of Treg cells (10^6)
wt
Fxop3cre;Rbx1fl/fl
c
wt
Foxp3cre;Rbx1fl/fl
45.3
12.8
Ki67-APC
Foxp3-PE
p=0.0002
Ki67hi/Treg (%)
wt
Fxop3cre;Rbx1fl/fl
d
wt
Fxop3cre;Rbx1fl/fl
0.659
5.19
Active Casp3-PE
Foxp3-A488
p=0.0282
active Casp3+/Treg (%)
wt
Fxop3cre;Rbx1fl/fl

Supplementary Figure 7. Impaired proliferation and elevated apoptosis of Rbx1-deficient Treg cells

(a) The proportion of Treg cells among $CD4^+$-T cells from peripheral lymph nodes of *wt* and $Foxp3^{cre}$;$Rbx1^{fl/fl}$ mice (p23 for representative image; p19-23, *n* =7 biologically independent samples from both male and female mice, p<0.0001 for statistics analysis).

(b) Treg cell numbers in peripheral lymph nodes from *wt* and $Foxp3^{cre}$;$Rbx1^{fl/fl}$ mice (p19-23, *n*=4 biologically independent samples from both male and female mice).

(c) Expression of Ki67 in Treg cells from peripheral lymph nodes of *wt* and $Foxp3^{cre}$;$Rbx1^{fl/fl}$ mice (p19 for representative image; p19-23, *n* =5 biologically independent samples for statistics analysis from both male and female mice).

(d) Active Casp3 in Treg cells from peripheral lymph nodes of *wt* and $Foxp3^{cre}$;$Rbx1^{fl/fl}$ mice (p19 for representative image; p19-23, *n* =4 biologically independent samples for statistics analysis from both male and female mice).

All error bars represent the SEM, data are presented as mean values +/- SEM. The *p* values were calculated by Mann–Whitney test. Source data are provided as a Source Data file.

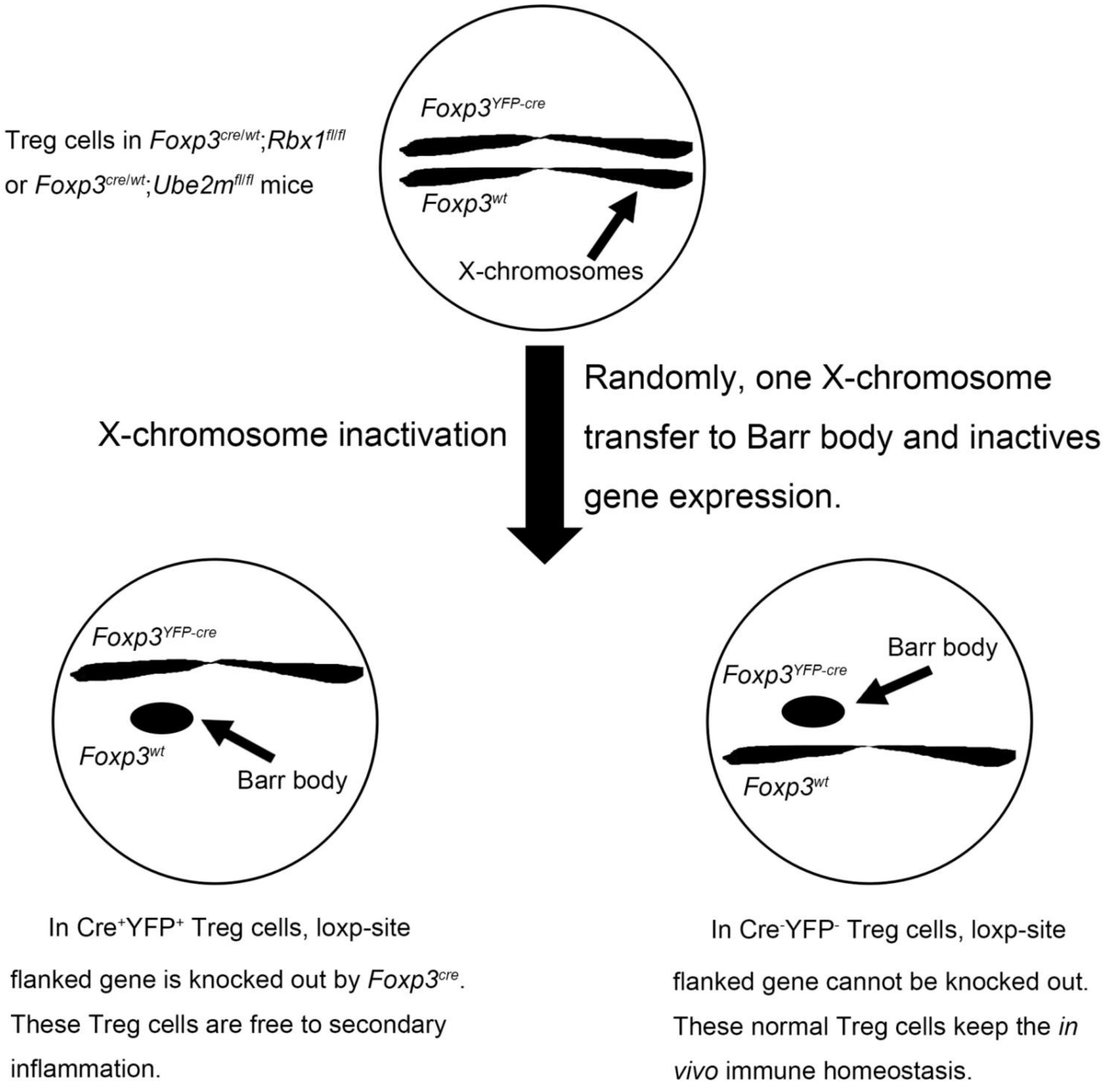

Supplementary Figure 8. Schematic demonstration how random X-chromosome inactivation protects female *Foxp3*$^{cre/wt}$;*Rbx1*$^{fl/fl}$ (or *Foxp3*$^{cre/wt}$;*Ube2m*$^{fl/fl}$) mice from inflammation

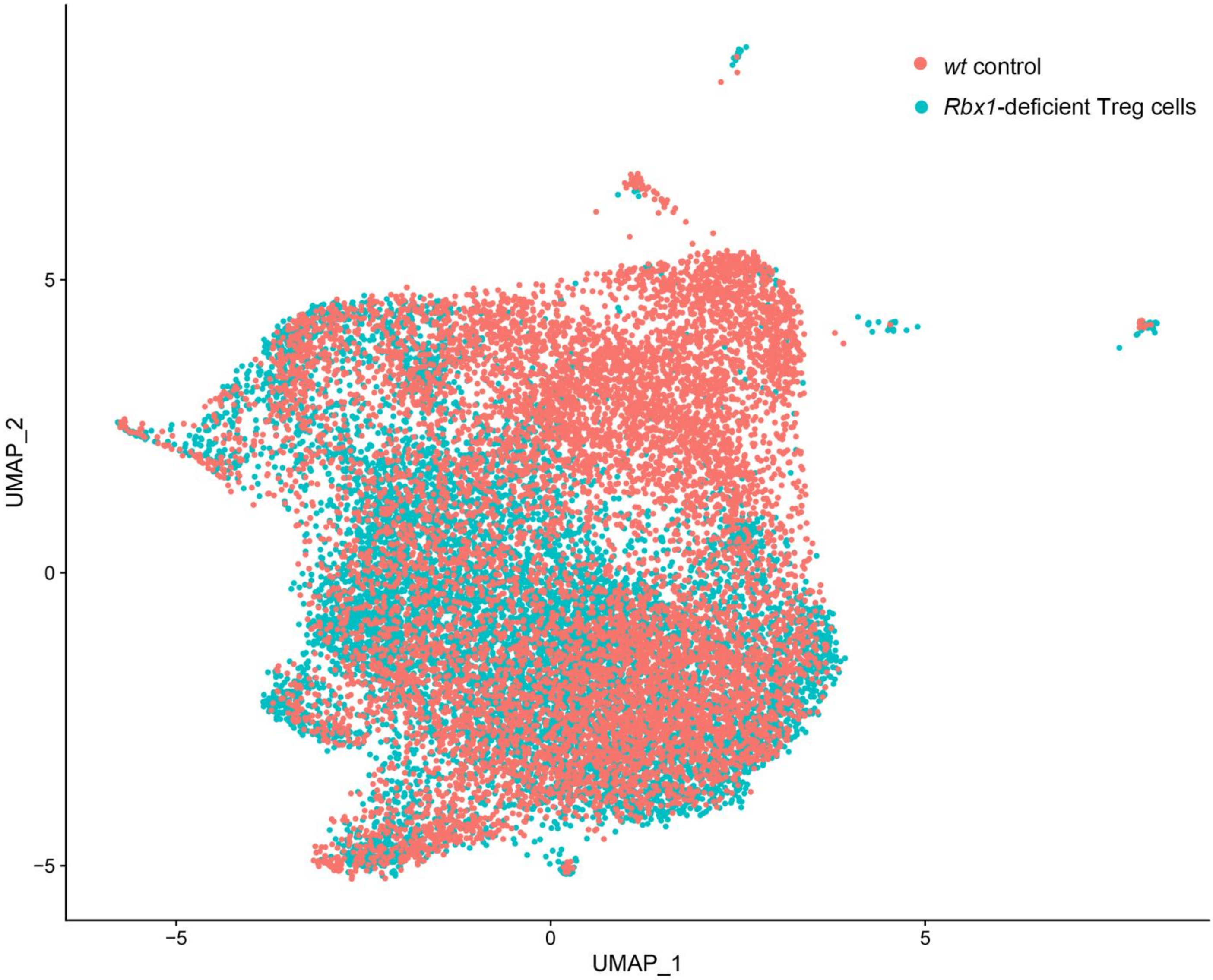

Supplementary Figure 9. The distribution of *wt* and *Rbx1*-deficient Treg cells in UMAP visualization

The clusters of *wt* and *Rbx1*-deficient Treg cells were largely overlapped with the exception of Cluster 0, 3, 4, as also shown in Fig. 3a.

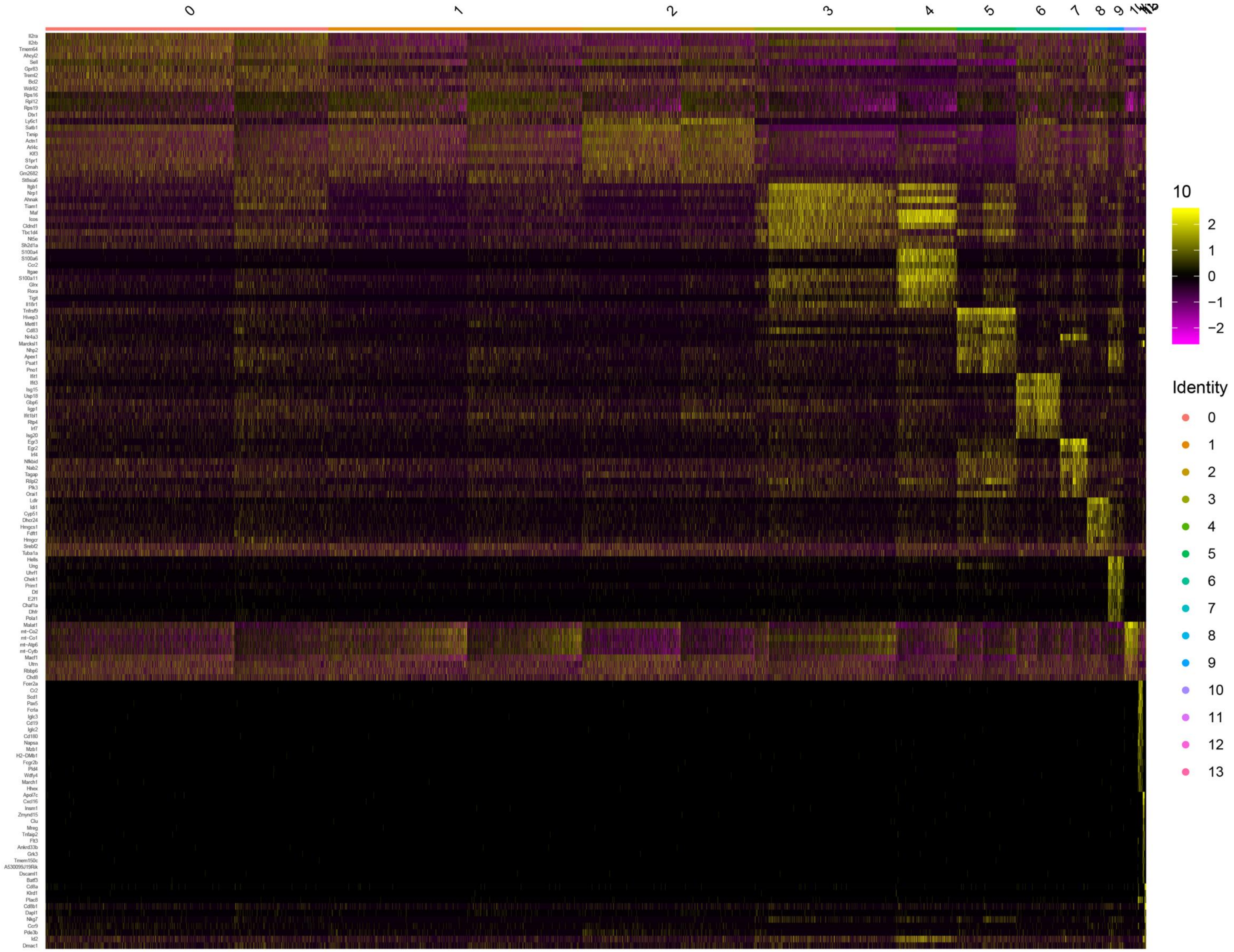
0
1
2
3
4
5
6
7
8
9
Il2ra
Il2rb
Tmem64
Ahcyl2
Sell
Gpr83
Treml2
Bcl2
Wdr82
Rps16
Rpl12
Rps19
Dtx1
Ly6c1
Satb1
Txnip
Actn1
Arl4c
Klf3
S1pr1
Cmah
Gm2682
St8sia6
Itgb1
Nrp1
Ahnak
Tiam1
Maf
Icos
Cldnd1
Tbc1d4
Nt5e
Sh2d1a
S100a4
S100a6
Ccr2
Itgae
S100a11
Glrx
Rora
Tigit
Il18r1
Tnfrsf9
Hivep3
Mettl1
Cd83
Nr4a3
Marcksl1
Nhp2
Apex1
Psat1
Pno1
Ifit1
Ifit3
Isg15
Usp18
Gbp6
Iigp1
Ifit1bl1
Rtp4
Irf7
Isg20
Egr3
Egr2
Irf4
Nfkbid
Nab2
Tagap
Rilpl2
Plk3
Orai1
Ldlr
Idi1
Cyp51
Dhcr24
Hmgcs1
Fdft1
Hmgcr
Srebf2
Tuba1a
Hells
Ung
Uhrf1
Chek1
Prim1
Dtl
E2f1
Chaf1a
Dhfr
Pola1
Malat1
mt−Co2
mt−Co1
mt−Atp6
mt−Cytb
Macf1
Utrn
Rbbp6
Chd8
Fcer2a
Cr2
Scd1
Pax5
Fcrla
Iglc3
Cd19
Iglc2
Cd180
Napsa
Mzb1
H2−DMb1
Fcgr2b
Pld4
Wdfy4
March1
Hhex
Apol7c
Cxcl16
Insm1
Zmynd15
Clu
Mreg
Tnfaip2
Flt3
Ankrd33b
Grk3
Tmem150c
A530099J19Rik
Dscaml1
Batf3
Cd8a
Klrd1
Plac8
Cd8b1
Dapl1
Nkg7
Ccr9
Pde3b
Id2
Dmac1
10
2
1
0
−1
−2
Identity
0
1
2
3
4
5
6
7
8
9
10
11
12
13

Supplementary Figure 10. Heat map of the characteristic genes of sub-populations in *wt* and *Rbx1*-deficient Treg cells

Top 10 characteristic genes of each of 14 clusters were presented.

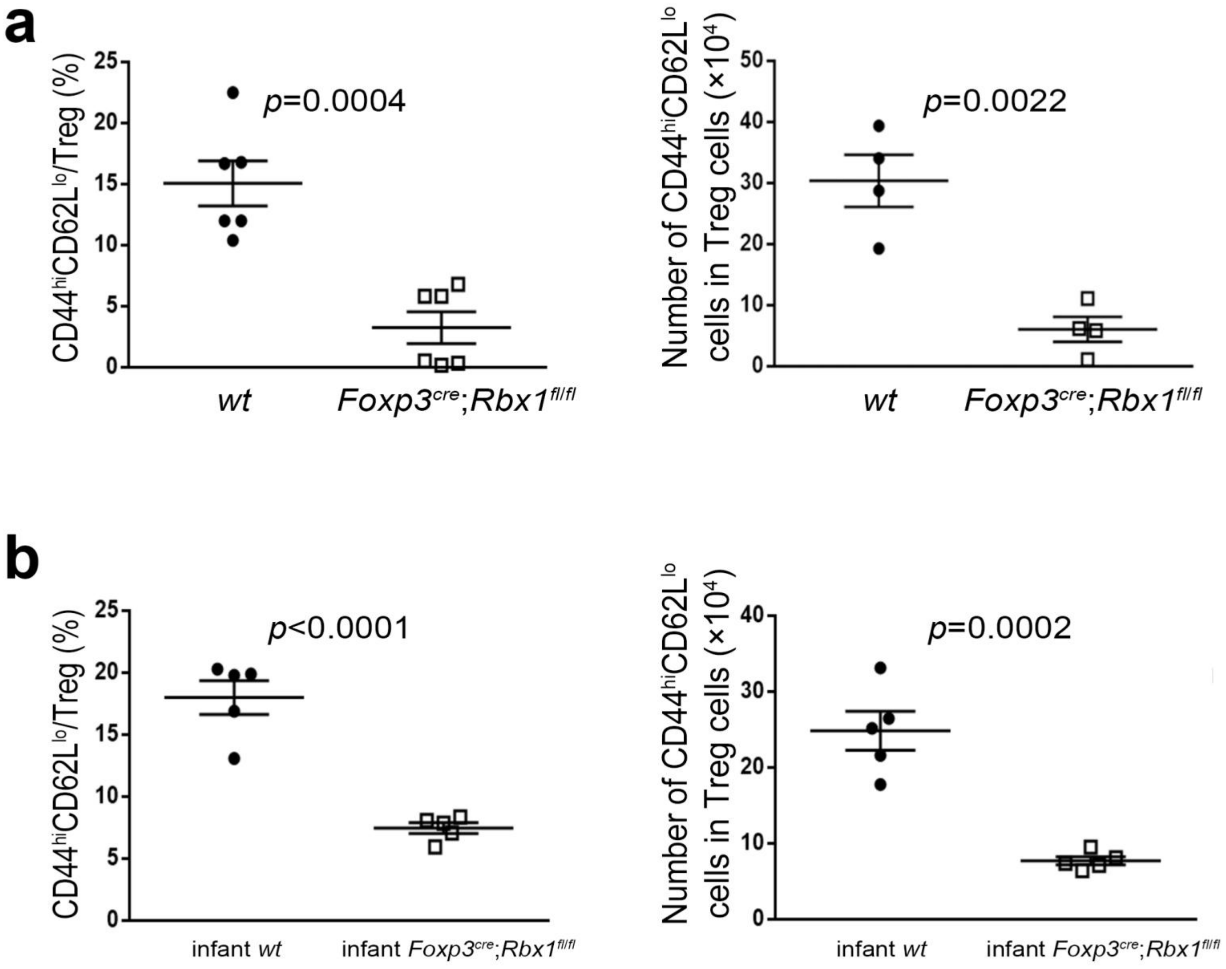

Supplementary Figure 11. FACS-based analysis of the Treg cell subpopulations in the lymph nodes of *wt* vs. *Foxp3^{cre}*;*Rbx1$^{fl/fl}$* mice at two different ages

(a) The proportion and number of CD44^{hi}CD62^{lo} effector/memory cells among Treg cells from peripheral lymph nodes of *wt* and *Foxp3^{cre}*;*Rbx1$^{fl/fl}$* mice (p19-23, *n* =6 or 4 biologically independent samples respectively from both male and female mice).

(b) The proportion and number of CD44^{hi}CD62^{lo} effector/memory cells among Treg cells from peripheral lymph nodes of *wt* and *Foxp3^{cre}*;*Rbx1$^{fl/fl}$* mice (p8, *n* =5 biologically independent samples from both male and female mice, p<0.0001(left) and p=0.0002 (right)).

All error bars represent the SEM, data are presented as mean values +/- SEM. The *p* values were calculated by Mann–Whitney test. Source data are provided as a Source Data file.

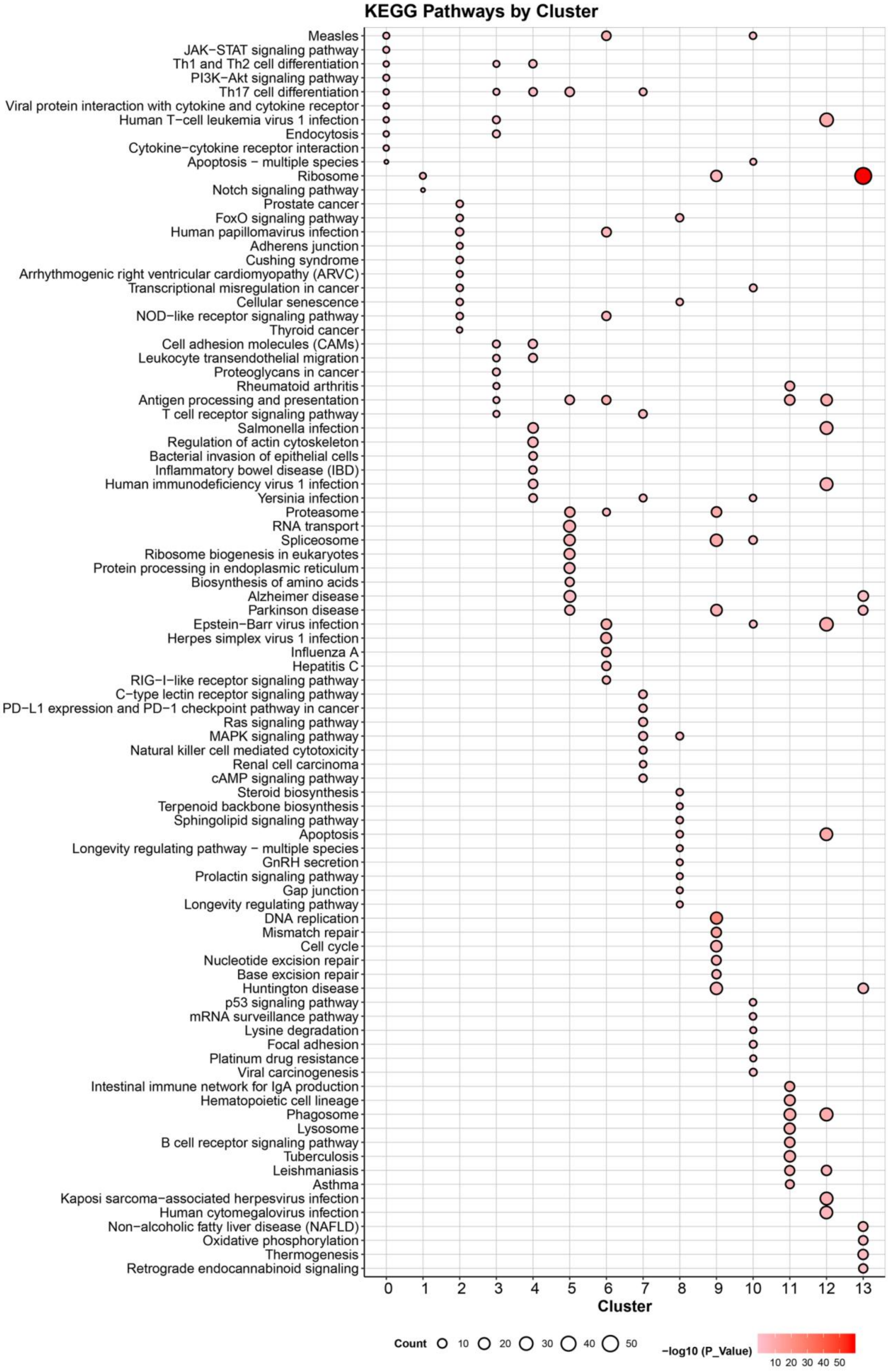

Supplementary Figure 12. KEGG pathway enrichment of clusters derived from scRNA-seq analysis of *wt* and *Rbx1*-deficient Treg cells

Top 10 altered pathways of 14 clusters were presented.

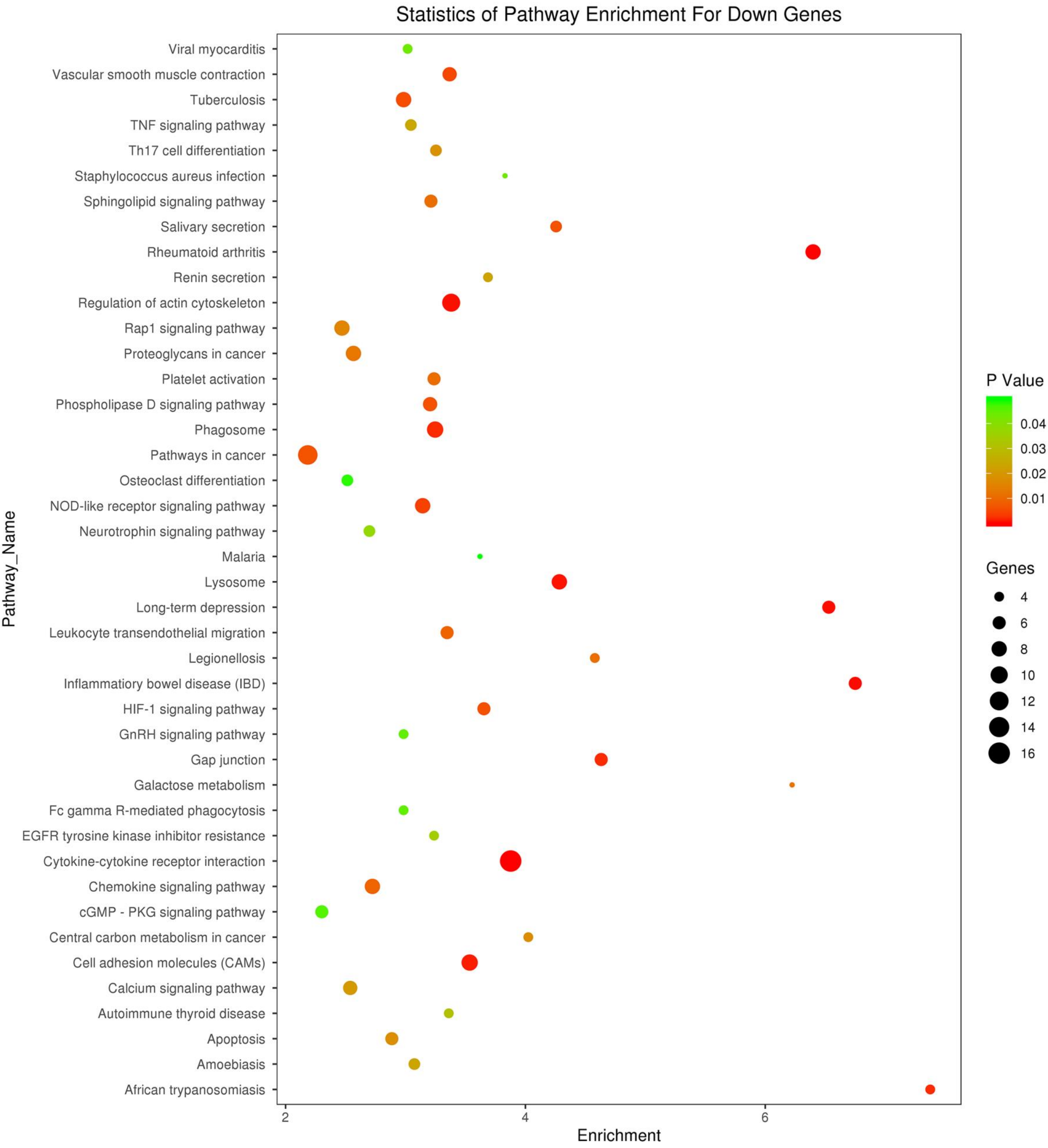

Statistics of Pathway Enrichment For Down Genes
Viral myocarditis
Vascular smooth muscle contraction
Tuberculosis
TNF signaling pathway
Th17 cell differentiation
Staphylococcus aureus infection
Sphingolipid signaling pathway
Salivary secretion
Rheumatoid arthritis
Renin secretion
Regulation of actin cytoskeleton
Rap1 signaling pathway
Proteoglycans in cancer
Platelet activation
Phospholipase D signaling pathway
Phagosome
Pathways in cancer
Osteoclast differentiation
NOD-like receptor signaling pathway
Neurotrophin signaling pathway
Malaria
Lysosome
Long-term depression
Leukocyte transendothelial migration
Legionellosis
Inflammatiory bowel disease (IBD)
HIF-1 signaling pathway
GnRH signaling pathway
Gap junction
Galactose metabolism
Fc gamma R-mediated phagocytosis
EGFR tyrosine kinase inhibitor resistance
Cytokine-cytokine receptor interaction
Chemokine signaling pathway
cGMP - PKG signaling pathway
Central carbon metabolism in cancer
Cell adhesion molecules (CAMs)
Calcium signaling pathway
Autoimmune thyroid disease
Apoptosis
Amoebiasis
African trypanosomiasis
Pathway_Name
2
4
6
Enrichment
P Value
0.04
0.03
0.02
0.01
Genes
4
6
8
10
12
14
16

Supplementary Figure 13. Downregulated pathways in CD4^{+}YFP^{+} Treg cells from female *Foxp3$^{cre/wt}$* and *Foxp3$^{cre/wt}$;Rbx1$^{fl/fl}$* mice (8-10 weeks old), determined by transcriptional profiling

The genes with alters more than 2-folds, with a *p*-value<0.05 were selected to KEGG analysis.

p values of pathways in the figure: Viral myocarditis, *p*=0.0434; Vascular smooth muscle contraction, *p*=0.0047; Tuberculosis, *p*=0.0053; TNF signaling pathway, *p*=0.0241; Th17 cell differentiation, *p*=0.0187; Staphylococcus aureus infection, *p*=0.0432; Sphingolipid signaling pathway, *p*=0.0109; Salivary secretion, *p*=0.0062; Rheumatoid arthritis, *p*<0.0001; Renin secretion, *p*=0.0230; Regulation of actin cytoskeleton, *p*=0.0004; Rap1 signaling pathway, *p*=0.0157; Proteoglycans in cancer, *p*=0.0127; Platelet activation, *p*=0.0105; Phospholipase D signaling pathway, *p*=0.0061; Phagosome, *p*=0.0018; Pathways in cancer, *p*=0.0063; Osteoclast differentiation, *p*=0.0488; NOD-like receptor signaling pathway, *p*=0.0038; Neurotrophin signaling pathway, *p*=0.0378; Malaria, *p*=0.0497; Lysosome, *p*=0.0005; Long-term depression, *p*=0.0003; Leukocyte transendothelial migration, *p*=0.0090; Legionellosis, *p*=0.0111; Inflammatiory bowel disease (IBD), *p*=0.0002; HIF-1 signaling pathway, *p*=0.0059; GnRH signaling pathway, *p*=0.0450; Gap junction, *p*=0.0018; Galactose metabolism, *p*=0.0120; Fc gamma R-mediated phagocytosis, *p*=0.0450; EGFR tyrosine kinase inhibitor resistance, *p*=0.0349; Cytokine-cytokine receptor interaction, *p*<0.0001; Chemokine signaling pathway, *p*=0.0091; cGMP - PKG signaling pathway, *p*=0.0462; Central carbon metabolism in cancer, *p*=0.0172; Cell adhesion molecules (CAMs), *p*=0.0010; Calcium signaling pathway, *p*=0.0203; Autoimmune thyroid disease, *p*=0.0310; Apoptosis, *p*=0.0176; Amoebiasis, *p*=0.0233; African trypanosomiasis, *p*=0.0019.

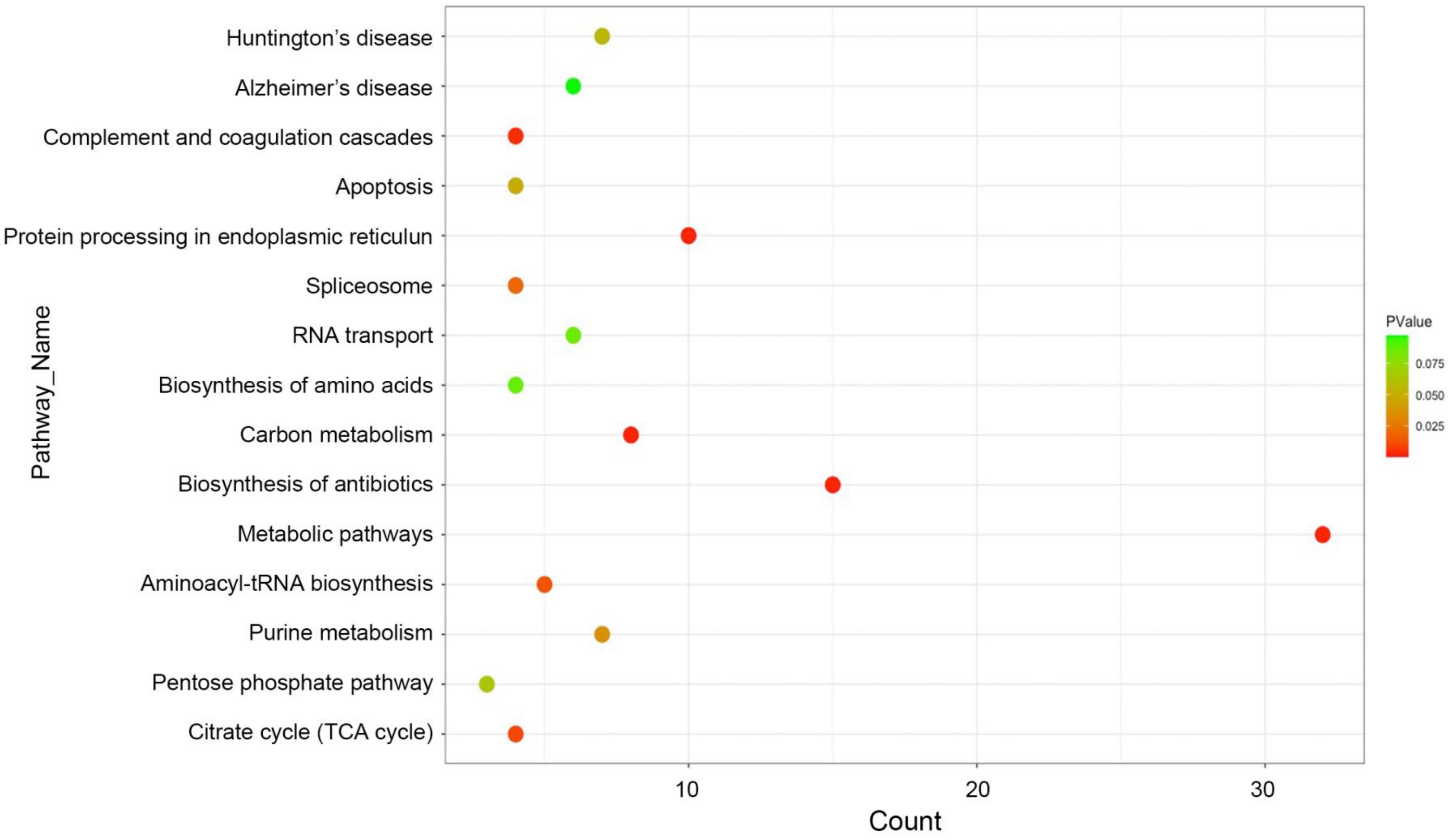

Supplementary Figure 14. Downregulated pathways in CD4$^+$YFP$^+$ Treg cells from female *Foxp3*$^{cre/wt}$ and *Foxp3*$^{cre/wt}$;*Rbx1*$^{fl/fl}$ mice (8-10 weeks old), determined by proteomic profiling

The proteins with alters more than 2-folds, with a *p*-value<0.05 were selected to KEGG analysis.

p values of pathways in the figure: Huntington’s disease, *p*=0.0553; Alzheimer’s disease, *p*=0.0971; Complement and coagulation cascades, *p*=0.0041; Apoptosis, 0.0495; Protein processing in endoplasmic reticulun, *p*=0.0005; Spliceosome, *p*=0.0189; RNA transport, *p*=0.0852; Biosynthesis of amino acids, *p*=0.0870; Carbon metabolism, *p*=0.0011; Biosynthesis of antibiotics, *p*<0.0001; Metabolic pathways, *p*=0.0007; Aminoacyl-tRNA biosynthesis, *p*=0.0128; Purine metabolism, *p*=0.0368; Pentose phosphate pathway, *p*=0.0638; Citrate cycle (TCA cycle), *p*=0.0094.

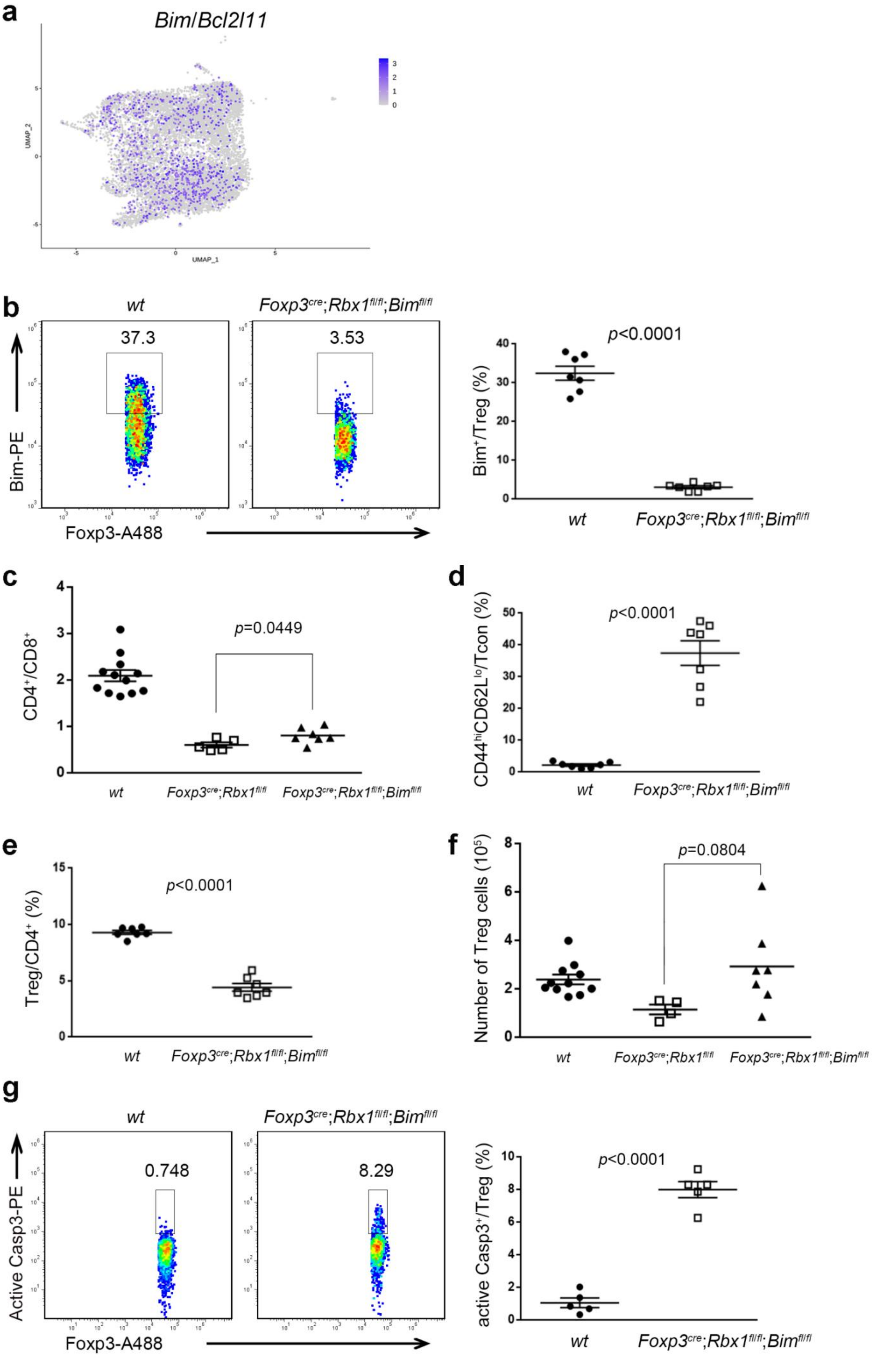
a
Bim/Bcl2l11
UMAP_2
UMAP_1
b
wt
Foxp3cre;Rbx1fl/fl;Bimfl/fl
37.3
3.53
Bim-PE
Foxp3-A488
p<0.0001
Bim+/Treg (%)
wt
Foxp3cre;Rbx1fl/fl;Bimfl/fl
c
p=0.0449
CD4+/CD8+
wt
Foxp3cre;Rbx1fl/fl
Foxp3cre;Rbx1fl/fl;Bimfl/fl
d
p<0.0001
CD44hiCD62Llo/Tcon (%)
wt
Foxp3cre;Rbx1fl/fl;Bimfl/fl
e
p<0.0001
Treg/CD4+ (%)
wt
Foxp3cre;Rbx1fl/fl;Bimfl/fl
f
p=0.0804
Number of Treg cells (105)
wt
Foxp3cre;Rbx1fl/fl
Foxp3cre;Rbx1fl/fl;Bimfl/fl
g
wt
Foxp3cre;Rbx1fl/fl;Bimfl/fl
0.748
8.29
Active Casp3-PE
Foxp3-A488
p<0.0001
active Casp3+/Treg (%)
wt
Foxp3cre;Rbx1fl/fl;Bimfl/fl

Supplementary Figure 15. Deletion of Bim partially rescue the phenotype of *Foxp3*^{cre};*Rbx1*$^{fl/fl}$ mice

(a) Expression of *Bim*/*Bcl2l11* in Treg cells revealed by sc-RNA sequence.

(b) Bim in Treg cells from peripheral lymph nodes of *wt* and *Foxp3*^{cre};*Rbx1*$^{fl/fl}$;*Bim*$^{fl/fl}$ mice (p20 for representative image; p19-21, n =7 biologically independent samples for statistics analysis from both male and female mice, p<0.0001).

(c) $CD4^+/CD8^+$ ratios in peripheral lymph nodes from *wt*, *Foxp3*^{cre};*Rbx1*$^{fl/fl}$ and *Foxp3*^{cre};*Rbx1*$^{fl/fl}$;*Bim*$^{fl/fl}$ mice (p19-23, n=12, 5 or 7 biologically independent samples respectively from both male and female mice).

(d) The proportion of $CD44^{hi}CD62^{lo}$ effector/memory cells among Tcon cells in peripheral lymph nodes from *wt* and *Foxp3*^{cre};*Rbx1*$^{fl/fl}$;*Bim*$^{fl/fl}$ mice (p19-21, n=7 biologically independent samples from both male and female mice, p<0.0001).

(e) Treg/$CD4^+$ ratios in peripheral lymph nodes from *wt* and *Foxp3*^{cre};*Rbx1*$^{fl/fl}$;*Bim*$^{fl/fl}$ mice (p19-21, n=7 biologically independent samples from both male and female mice, p<0.0001).

(f) Treg cell numbers in peripheral lymph nodes from *wt*, *Foxp3*^{cre};*Rbx1*$^{fl/fl}$ and *Foxp3*^{cre};*Rbx1*$^{fl/fl}$;*Bim*$^{fl/fl}$ mice (p19-23, n=11, 4 or 7 biologically independent samples respectively from both male and female mice).

(g) Active Casp3 in Treg cells from peripheral lymph nodes of *wt* and *Foxp3*^{cre};*Rbx1*$^{fl/fl}$;*Bim*$^{fl/fl}$ mice (p20 for representative image; p19-21, n =5 biologically independent samples from both male and female mice, p<0.0001 for statistics analysis).

All error bars represent the SEM, data are presented as mean values +/- SEM. The p values were calculated by Mann–Whitney test. Source data are provided as a Source Data file.

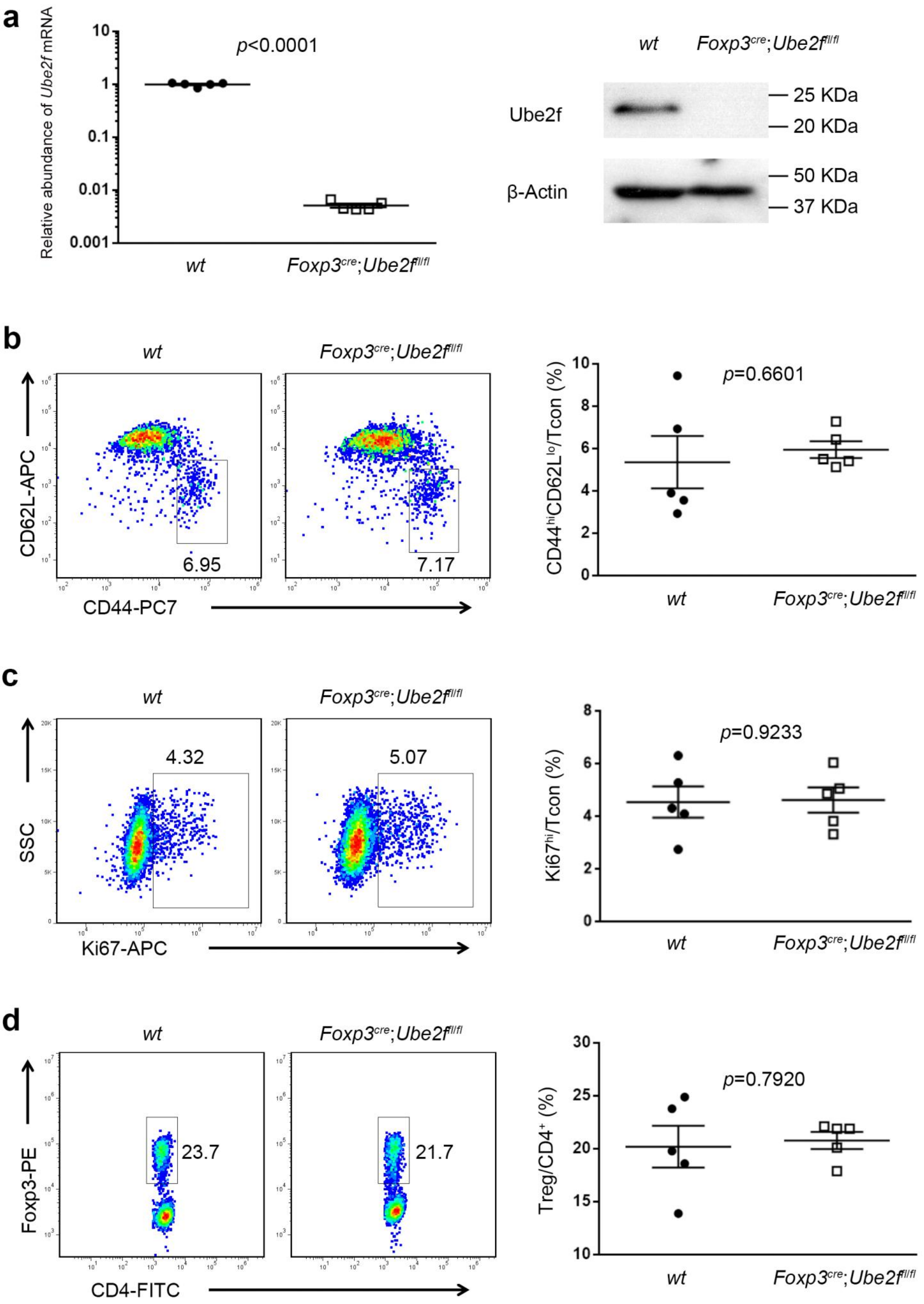

a
Relative abundance of Ube2f mRNA
10
1
0.1
0.01
0.001
p<0.0001
wt
Foxp3cre;Ube2ffl/fl
wt
Foxp3cre;Ube2ffl/fl
Ube2f
β-Actin
25 KDa
20 KDa
50 KDa
37 KDa
b
wt
Foxp3cre;Ube2ffl/fl
CD62L-APC
CD44-PC7
6.95
7.17
CD44hiCD62Llo/Tcon (%)
p=0.6601
wt
Foxp3cre;Ube2ffl/fl
c
wt
Foxp3cre;Ube2ffl/fl
SSC
Ki67-APC
4.32
5.07
Ki67hi/Tcon (%)
p=0.9233
wt
Foxp3cre;Ube2ffl/fl
d
wt
Foxp3cre;Ube2ffl/fl
Foxp3-PE
CD4-FITC
23.7
21.7
Treg/CD4+ (%)
p=0.7920
wt
Foxp3cre;Ube2ffl/fl

Supplementary Figure 16. Deficiency of Ube2f does not obviously impair Treg cell fitness at steady status

(a) Expression of *Ube2f* mRNA and Ube2f protein in $CD4^+YFP^+$ Treg cells from peripheral lymph nodes of *wt* and $Foxp3^{cre}$;$Ube2f^{fl/fl}$ mice (*n*=5 biologically independent samples from both male and female mice, p<0.0001).

(b) Expression of CD44 and CD62L in Tcon cells from peripheral lymph nodes of *wt* and $Foxp3^{cre}$;$Ube2f^{fl/fl}$ mice (16 weeks old, *n*=5 biologically independent samples from both male and female mice).

(c) Expression of Ki67 in Tcon cells in peripheral lymph nodes from *wt* and $Foxp3^{cre}$;$Ube2f^{fl/fl}$ mice (16 weeks old, *n*=5 biologically independent samples from both male and female mice).

(d) The proportion of Treg cells among $CD4^+$-T cells in peripheral lymph nodes from *wt* and $Foxp3^{cre}$;$Ube2f^{fl/fl}$ mice (16 weeks old, *n*=5 biologically independent samples from both male and female mice).

All error bars represent the SEM, data are presented as mean values +/- SEM. The *p* values were calculated by Mann–Whitney test. Source data are provided as a Source Data file.

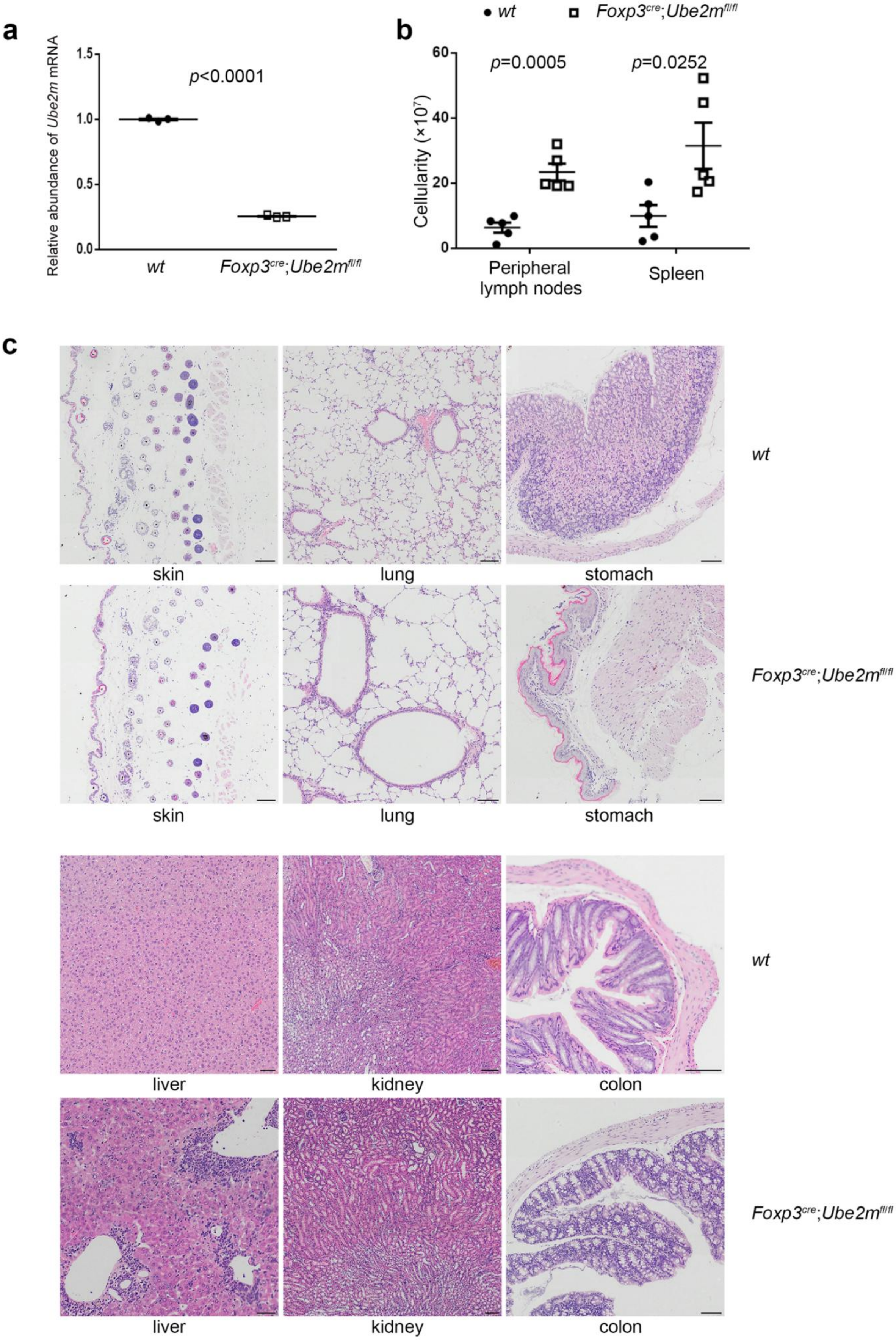
a
Relative abundance of Ube2m mRNA
1.5
1.0
0.5
0.0
p<0.0001
wt
Foxp3cre;Ube2mfl/fl
b
wt
Foxp3cre;Ube2mfl/fl
Cellularity (×10^7)
60
40
20
0
p=0.0005
p=0.0252
Peripheral lymph nodes
Spleen
c
skin
lung
stomach
wt
skin
lung
stomach
Foxp3cre;Ube2mfl/fl
liver
kidney
colon
wt
liver
kidney
colon
Foxp3cre;Ube2mfl/fl

Supplementary Figure 17. *Ube2m* deletion in Treg cells leads to inflammatory disorders in mice

(a) Expression of *Ube2m* mRNA in $CD4^+YFP^+$ Treg cells from peripheral lymph nodes of *wt* and $Foxp3^{cre}$;$Ube2m^{fl/fl}$ mice. Left, revealed by transcriptome analysis; right, revealed by q-PCR (n=3 biologically independent samples from both male and female mice, p<0.0001).

(b) Total cell numbers in peripheral lymph nodes and spleen from *wt* and $Foxp3^{cre}$;$Ube2m^{fl/fl}$ mice (16 weeks old, n=5 biologically independent samples from both male and female mice).

(c) H&E staining of skin, lung, stomach, liver, kidney, colon from *wt* and $Foxp3^{cre}$;$Ube2m^{fl/fl}$ mice (16 weeks old, male, scale bar = 50μm in liver, and 100μm in other organs, 3 times each experiment was repeated independently with similar results).

All error bars represent the SEM, data are presented as mean values +/- SEM. The *p* values were calculated by Mann–Whitney test. Source data are provided as a Source Data file.

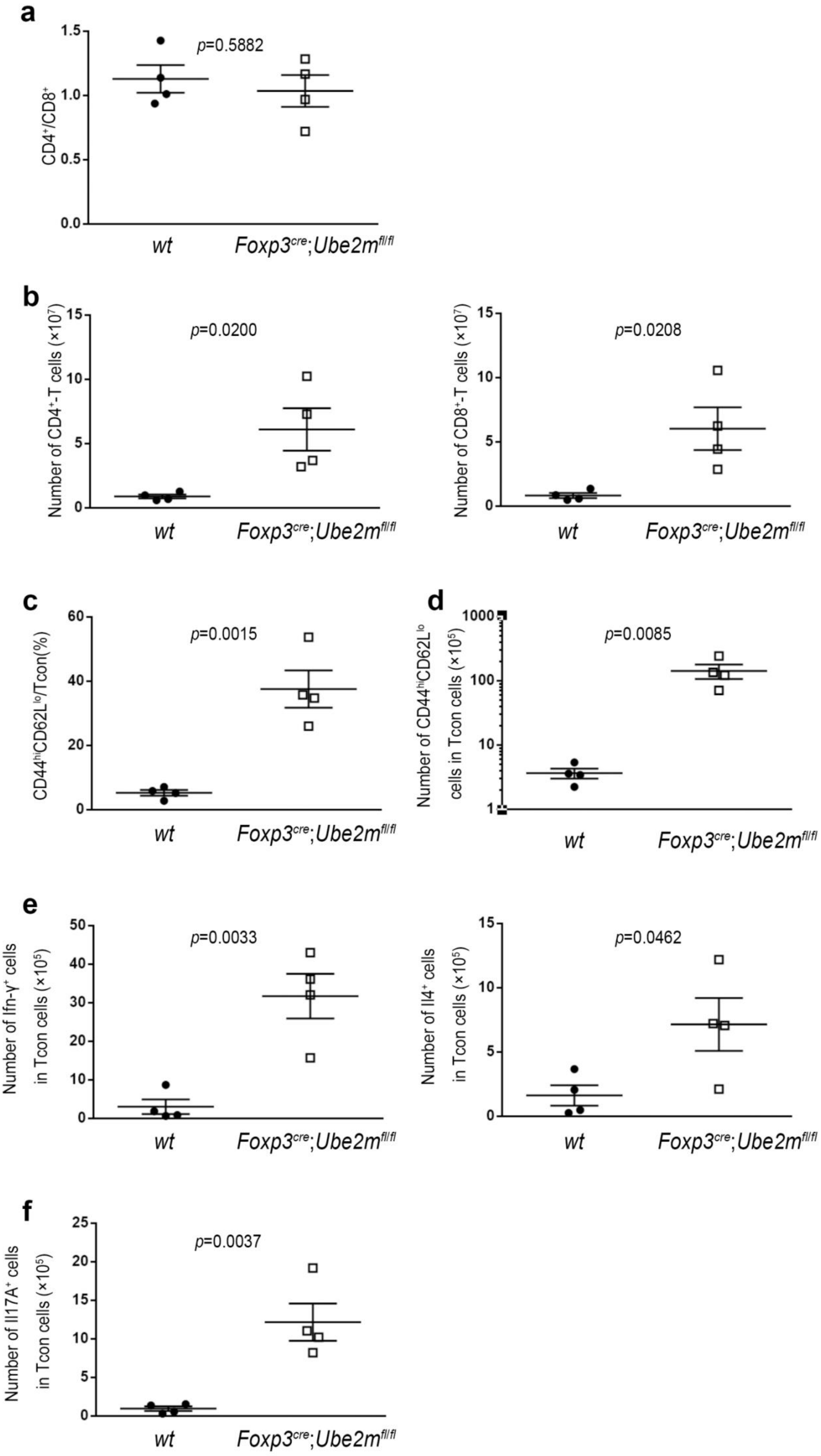

a
CD4+/CD8+
p=0.5882
1.5
1.0
0.5
0.0
wt
Foxp3cre;Ube2mfl/fl
b
Number of CD4+-T cells (×10^7)
p=0.0200
Number of CD8+-T cells (×10^7)
p=0.0208
15
10
5
0
wt
Foxp3cre;Ube2mfl/fl
c
CD44hiCD62Llo/Tcon(%)
p=0.0015
60
40
20
0
d
Number of CD44hiCD62Llo cells in Tcon cells (×10^5)
p=0.0085
1000
100
10
1
e
Number of Ifn-γ+ cells in Tcon cells (×10^5)
p=0.0033
50
40
30
20
10
0
Number of Il4+ cells in Tcon cells (×10^5)
p=0.0462
f
Number of Il17A+ cells in Tcon cells (×10^5)
p=0.0037
25
20
15
10
5
0
wt
Foxp3cre;Ube2mfl/fl

Supplementary Figure 18. Changes of ratios or numbers of immune cells in *Foxp3^cre^;Ube2m^fl/fl^* mice

(a) $CD4^+/CD8^+$ ratios in peripheral lymph nodes from *wt* and *$Foxp3^{cre}$;$Ube2m^{fl/fl}$* mice (16 weeks old, *n* =4 biologically independent samples from both male and female mice).

(b) Numbers of $CD4^+$ and $CD8^+$-T cells in peripheral lymph nodes from *wt* and *$Foxp3^{cre}$;$Ube2m^{fl/fl}$* mice (16 weeks old, *n* =4 biologically independent samples from both male and female mice).

(c) The proportion of $CD44^{hi}CD62^{lo}$ effector/memory cells among Tcon cells in peripheral lymph nodes from *wt* and *$Foxp3^{cre}$;$Ube2m^{fl/fl}$* mice (16 weeks old, *n* =4 biologically independent samples from both male and female mice).

(d) Number of $CD44^{hi}CD62L^{lo}$ cells in Tcon cells from peripheral lymph nodes of *wt* and *$Foxp3^{cre}$;$Ube2m^{fl/fl}$* mice (16 weeks old, *n* =4 biologically independent samples from both male and female mice).

(e) Number of Ifn-γ^+ and $Il4^+$ cells in Tcon cells from peripheral lymph nodes of *wt* and *$Foxp3^{cre}$;$Ube2m^{fl/fl}$* mice (16 weeks old, *n* =4 biologically independent samples from both male and female mice).

(f) Number of $Il17A^+$ cells in Tcon cells from peripheral lymph nodes of *wt* and *$Foxp3^{cre}$;$Ube2m^{fl/fl}$* mice (16 weeks old, *n* =4 biologically independent samples from both male and female mice).

All error bars represent the SEM, data are presented as mean values +/- SEM. The *p* values were calculated by Mann–Whitney test. Source data are provided as a Source Data file.

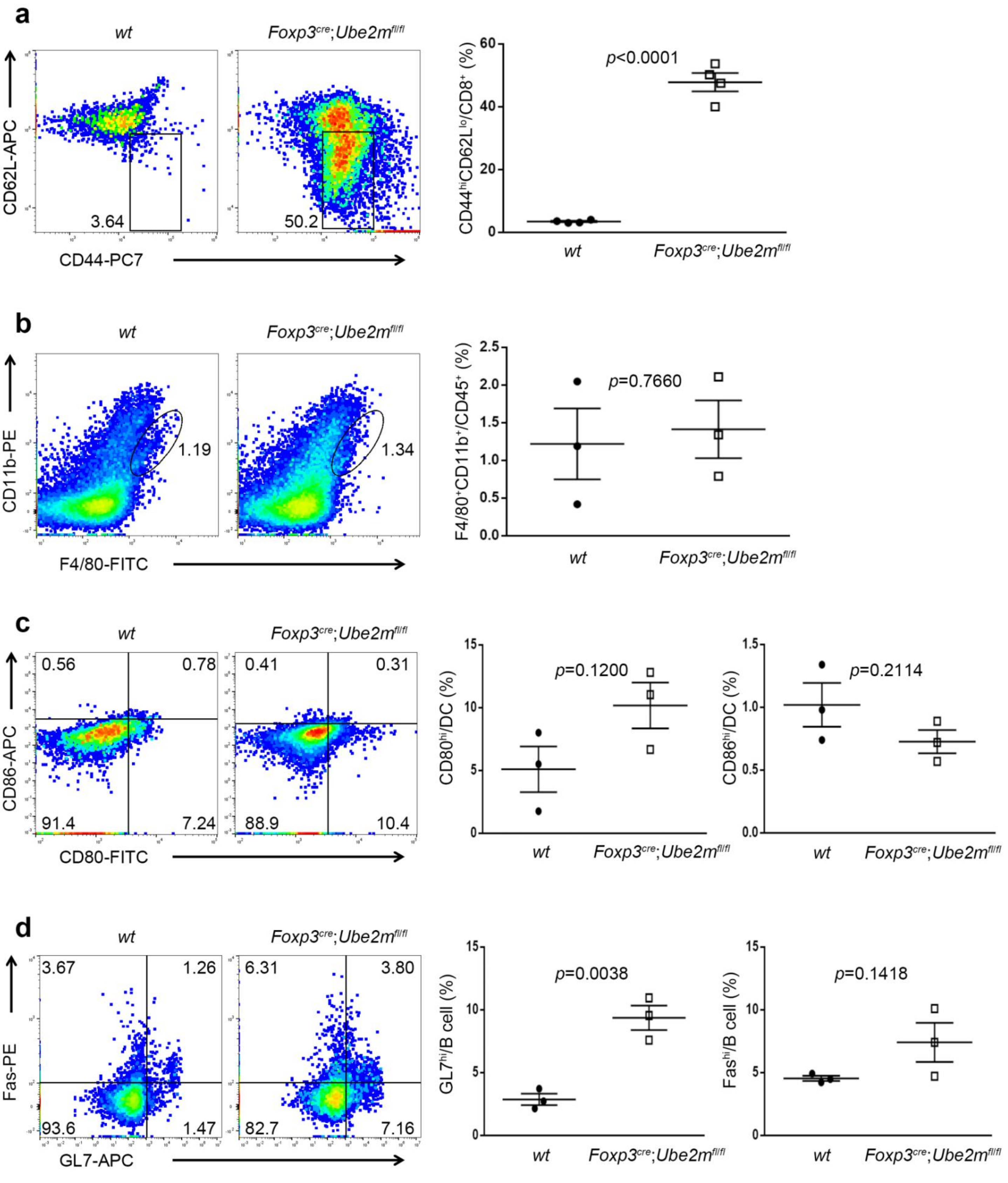
a
wt
Foxp3cre;Ube2mfl/fl
CD62L-APC
CD44-PC7
3.64
50.2
CD44hiCD62Llo/CD8+ (%)
p<0.0001
wt
Foxp3cre;Ube2mfl/fl
b
wt
Foxp3cre;Ube2mfl/fl
CD11b-PE
F4/80-FITC
1.19
1.34
F4/80+CD11b+/CD45+ (%)
p=0.7660
wt
Foxp3cre;Ube2mfl/fl
c
wt
Foxp3cre;Ube2mfl/fl
CD86-APC
CD80-FITC
0.56
0.78
91.4
7.24
0.41
0.31
88.9
10.4
CD80hi/DC (%)
p=0.1200
CD86hi/DC (%)
p=0.2114
wt
Foxp3cre;Ube2mfl/fl
d
wt
Foxp3cre;Ube2mfl/fl
Fas-PE
GL7-APC
3.67
1.26
93.6
1.47
6.31
3.80
82.7
7.16
GL7hi/B cell (%)
p=0.0038
Fashi/B cell (%)
p=0.1418
wt
Foxp3cre;Ube2mfl/fl

Supplementary Figure 19. Over-activation of multiple types of immune cells in *Foxp3*^{cre};*Ube2m*$^{fl/fl}$ mice

(a) Expression of CD44 and CD62L in CD8$^+$-T cells from peripheral lymph nodes of *wt* and *Foxp3*^{cre};*Ube2m*$^{fl/fl}$ mice (16 weeks old, n=4 biologically independent samples from both male and female mice, p<0.0001).

(b) Percentage of macrophages (F4/80$^+$CD11b$^+$) among CD45$^+$ cells from peripheral lymph nodes of *wt* and *Foxp3*^{cre};*Ube2m*$^{fl/fl}$ mice (16 weeks old, n=3 biologically independent samples from both male and female mice).

(c) Expression of CD80 and CD86 in dendritic cells from peripheral lymph nodes of *wt* and *Foxp3*^{cre};*Ube2m*$^{fl/fl}$ mice (16 weeks old, n=3 biologically independent samples from both male and female mice).

(d) Expression of GL7 and Fas in B cells from peripheral lymph nodes of *wt* and *Foxp3*^{cre};*Ube2m*$^{fl/fl}$ mice (16 weeks old, n=3 biologically independent samples from both male and female mice).

All error bars represent the SEM, data are presented as mean values +/- SEM. The p values were calculated by Mann–Whitney test. Source data are provided as a Source Data file.

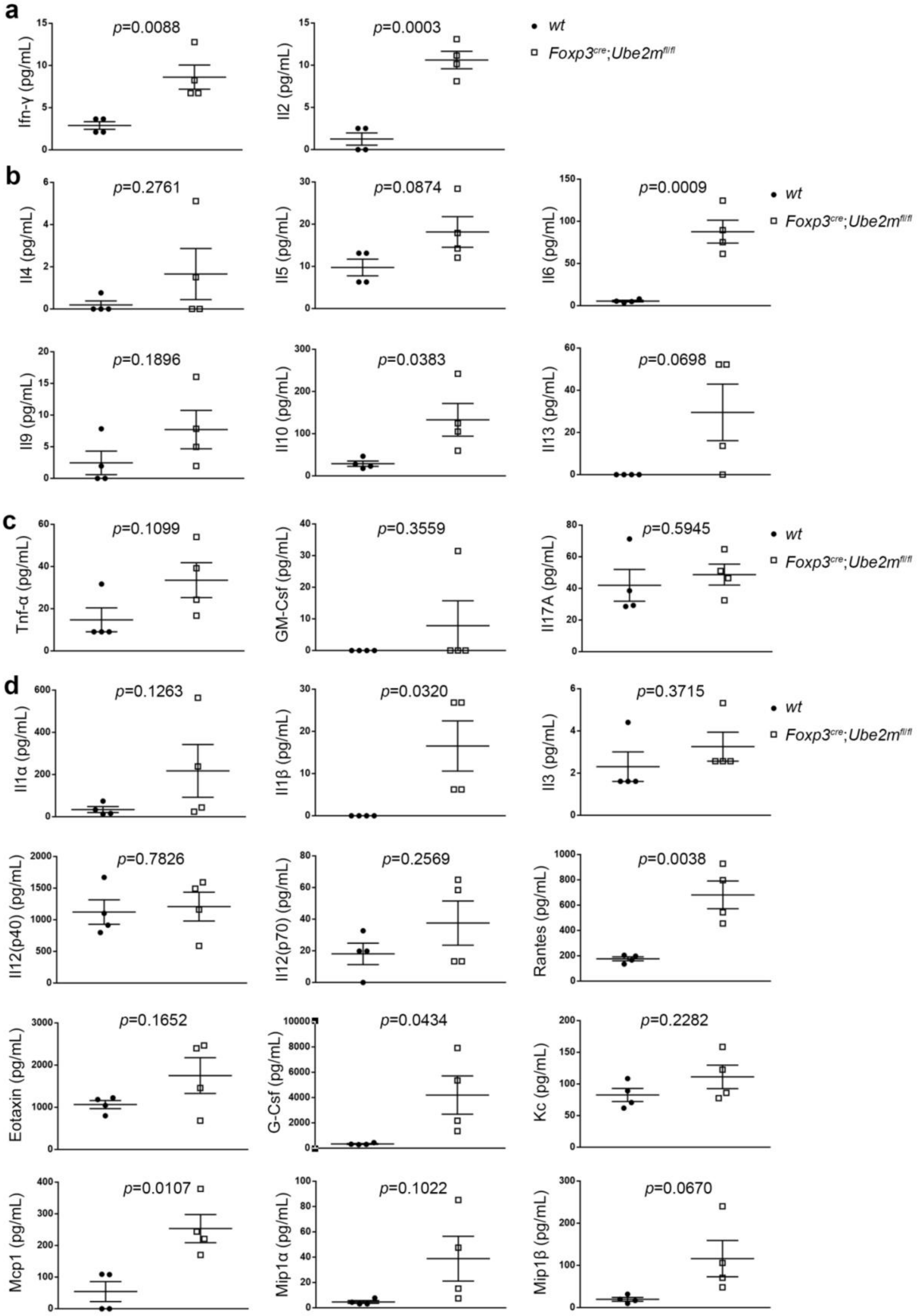

Supplementary Figure 20. Quantification of serum cytokines in *wt* and *Foxp3^{cre}*;*Ube2m$^{fl/fl}$* mice (16 weeks old, *n* =4 biologically independent samples from both male and female mice)

(a) T_H1 cytokines. **(b)** T_H2 cytokines. **(c)** T_H17 cytokines. **(d)** Other cytokines.

All error bars represent the SEM, data are presented as mean values +/- SEM. The *p* values were calculated by Mann–Whitney test. Source data are provided as a Source Data file.

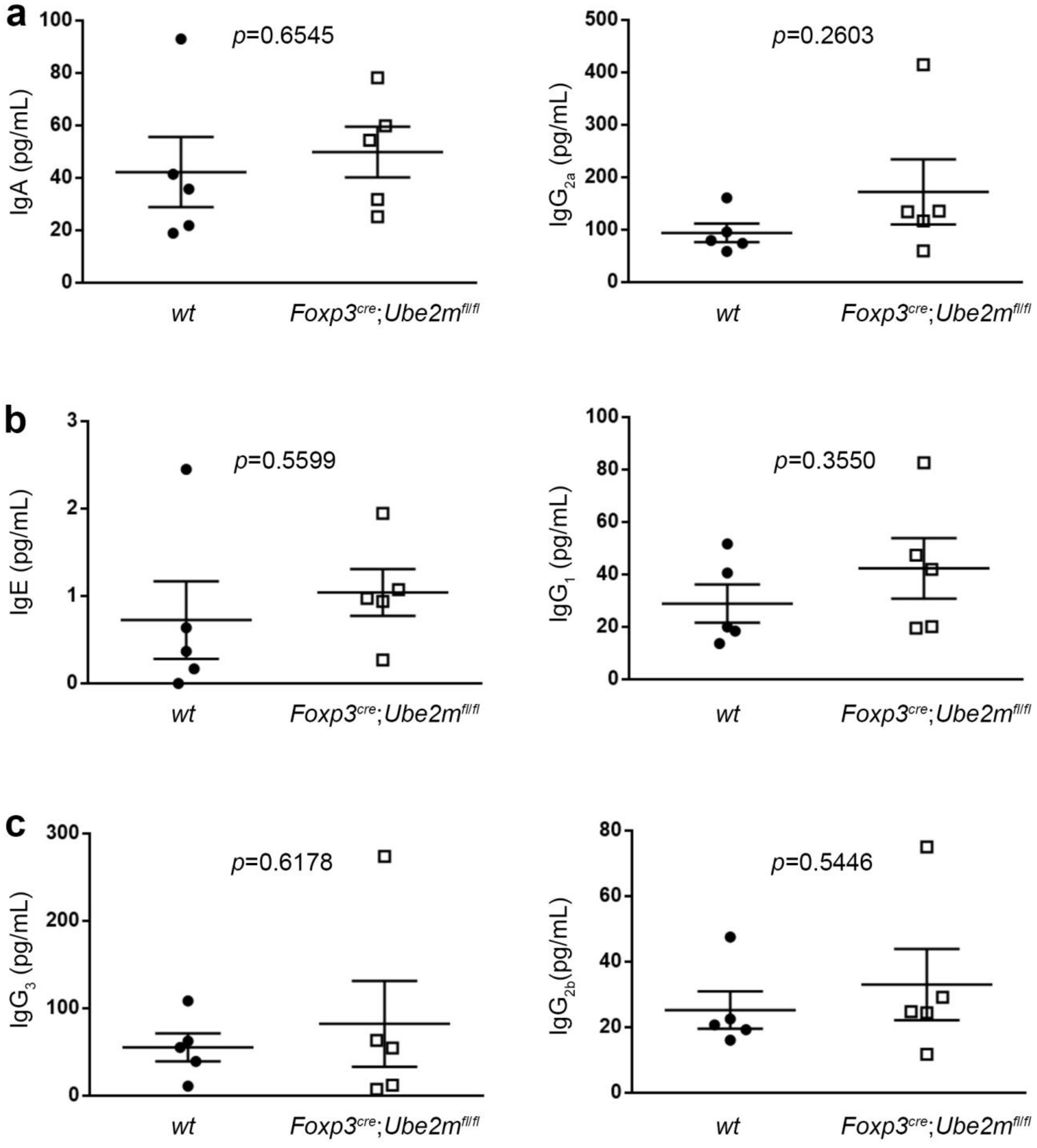

Supplementary Figure 21. Quantification of serum immunoglobulin subclasses in *wt* and *Foxp3*^{cre};*Ube2m*$^{fl/fl}$ mice (16 weeks old, *n* =5 biologically independent samples from both male and female mice)

(a) T_H1 antibodies. **(b)** T_H2 antibodies. **(c)** T_H17 antibodies.

All error bars represent the SEM, data are presented as mean values +/- SEM. The *p* values were calculated by Mann–Whitney test. Source data are provided as a Source Data file.

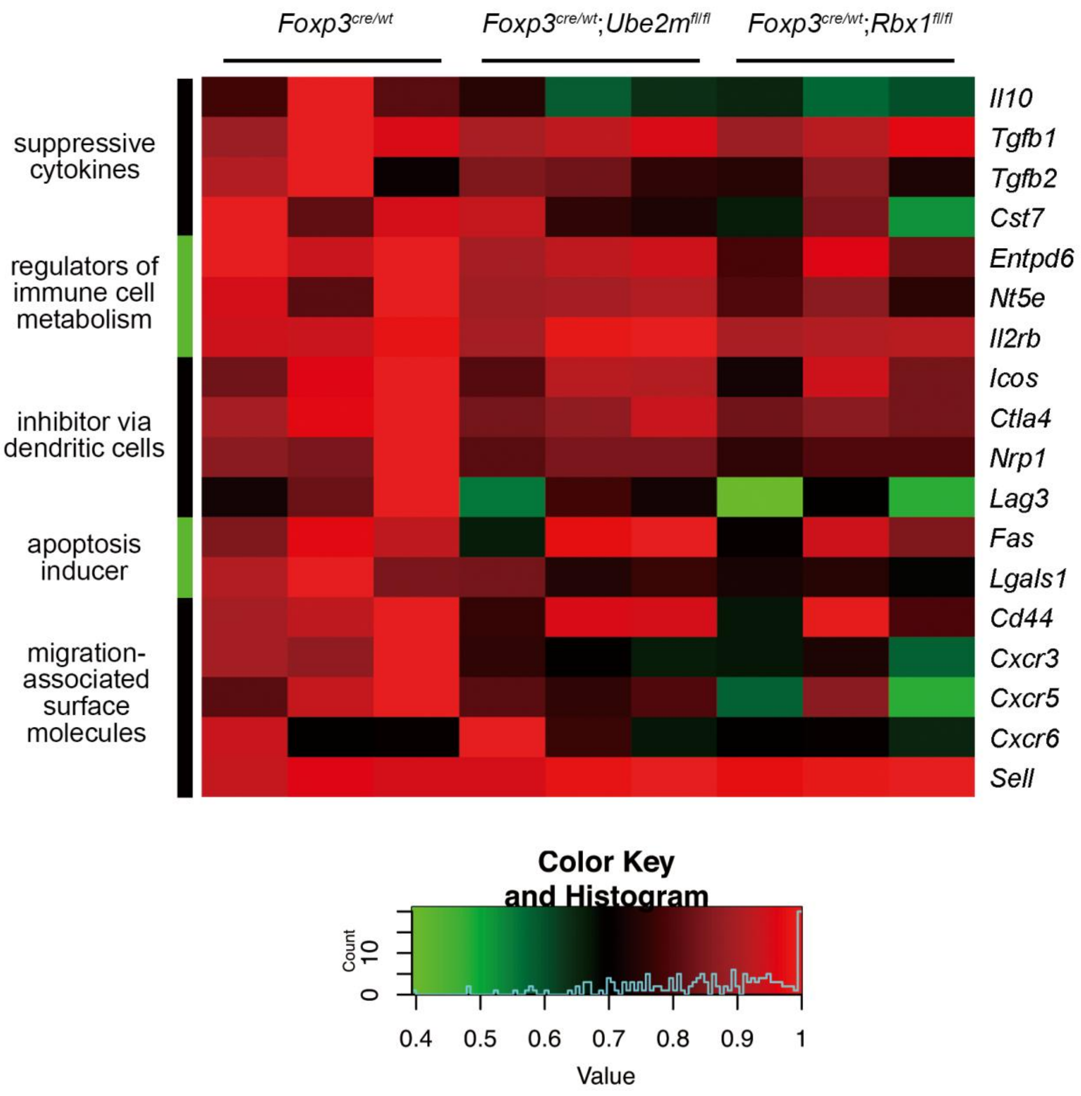

Supplementary Figure 22. The heat map derived from transcriptome profiling of Treg cells from three indicated genotypes with functional allocations.

The expression levels of indicated genes related to the Treg functions in CD4$^+$YFP$^+$ Treg cells derived from female *Foxp3$^{cre/wt}$*, *Foxp3$^{cre/wt}$;Ube2m$^{fl/fl}$* and *Foxp3$^{cre/wt}$;Rbx1$^{fl/fl}$* mice (8-10 weeks old), determined by transcriptional profiling.

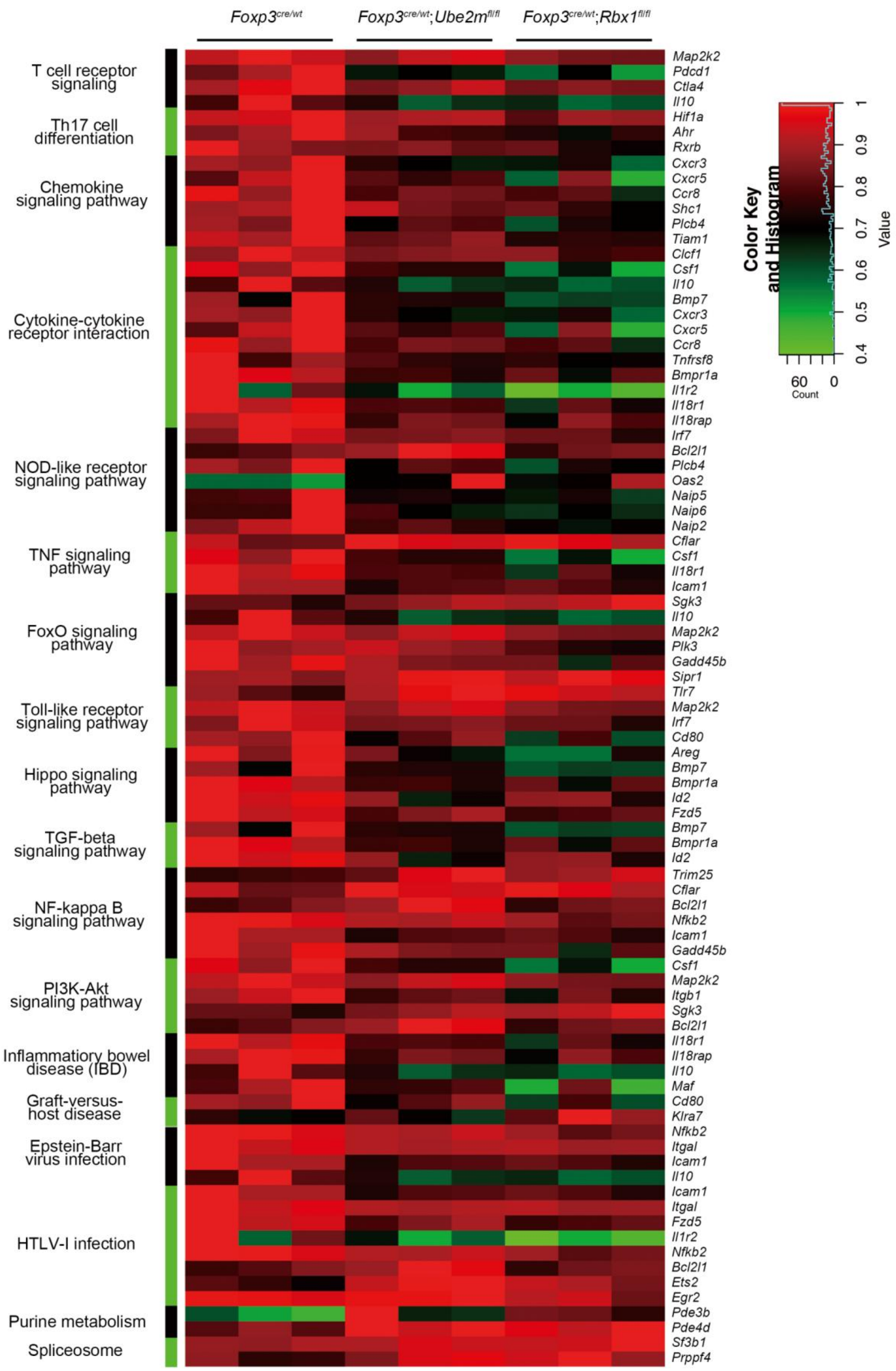

Supplementary Figure 23. The heat map derived from transcriptome profiling of Treg cells from three indicated genotypes with pathway allocations.

The expression levels of indicated genes and their associated signaling pathways in CD4$^+$YFP$^+$ Treg cells derived from female *Foxp3$^{cre/wt}$*, *Foxp3$^{cre/wt}$*;*Ube2m$^{fl/fl}$* and *Foxp3$^{cre/wt}$*;*Rbx1$^{fl/fl}$* mice (8-10 weeks old), determined by transcriptional profiling.

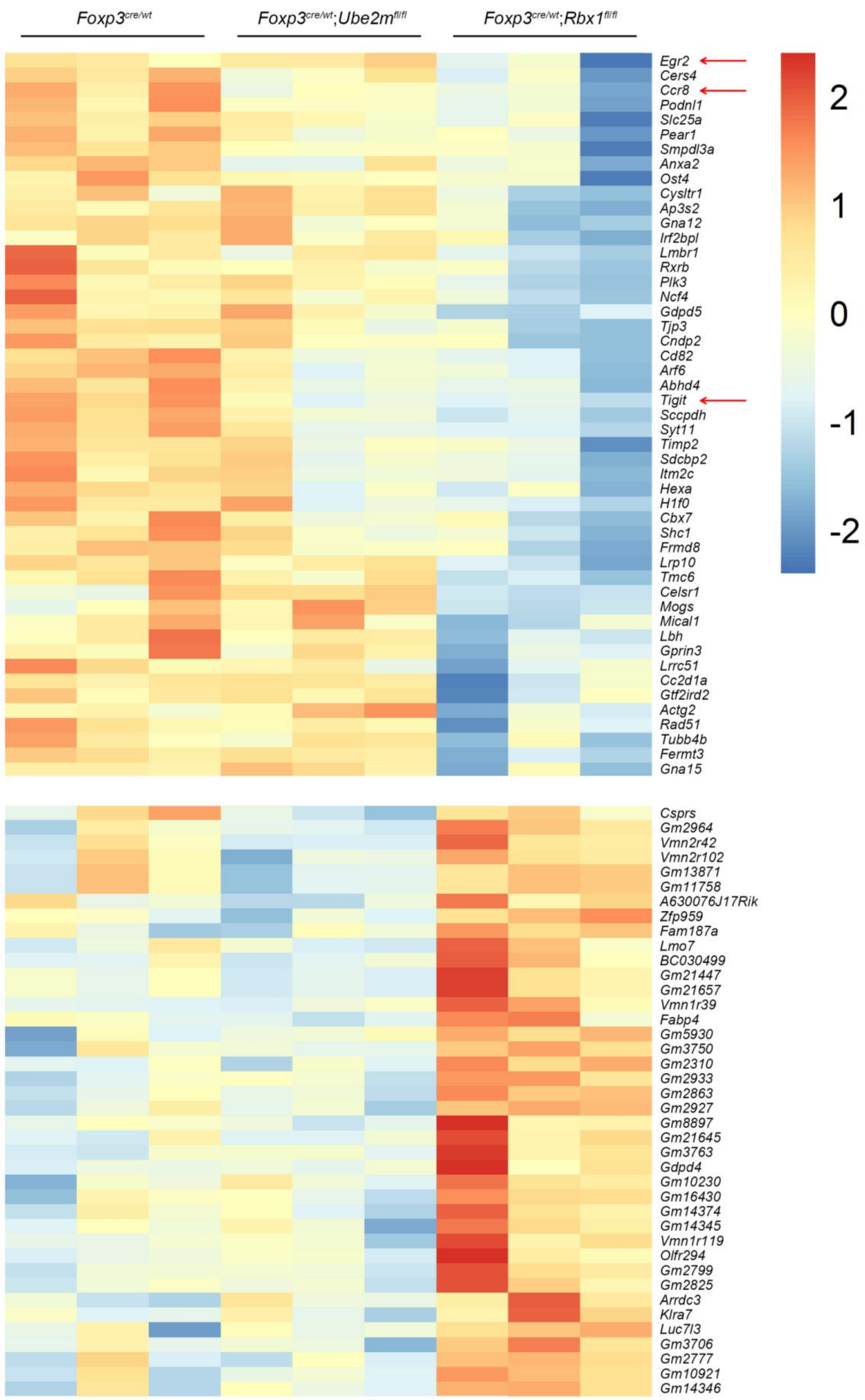

Supplementary Figure 24. Genes with altered expression selectively in *Rbx1*-Deficient Treg cells

Top portion: Genes with reduced expression;

Bottom panels: Genes with increased expression.

Three genes known to regulate Treg cell function were marked with arrows.

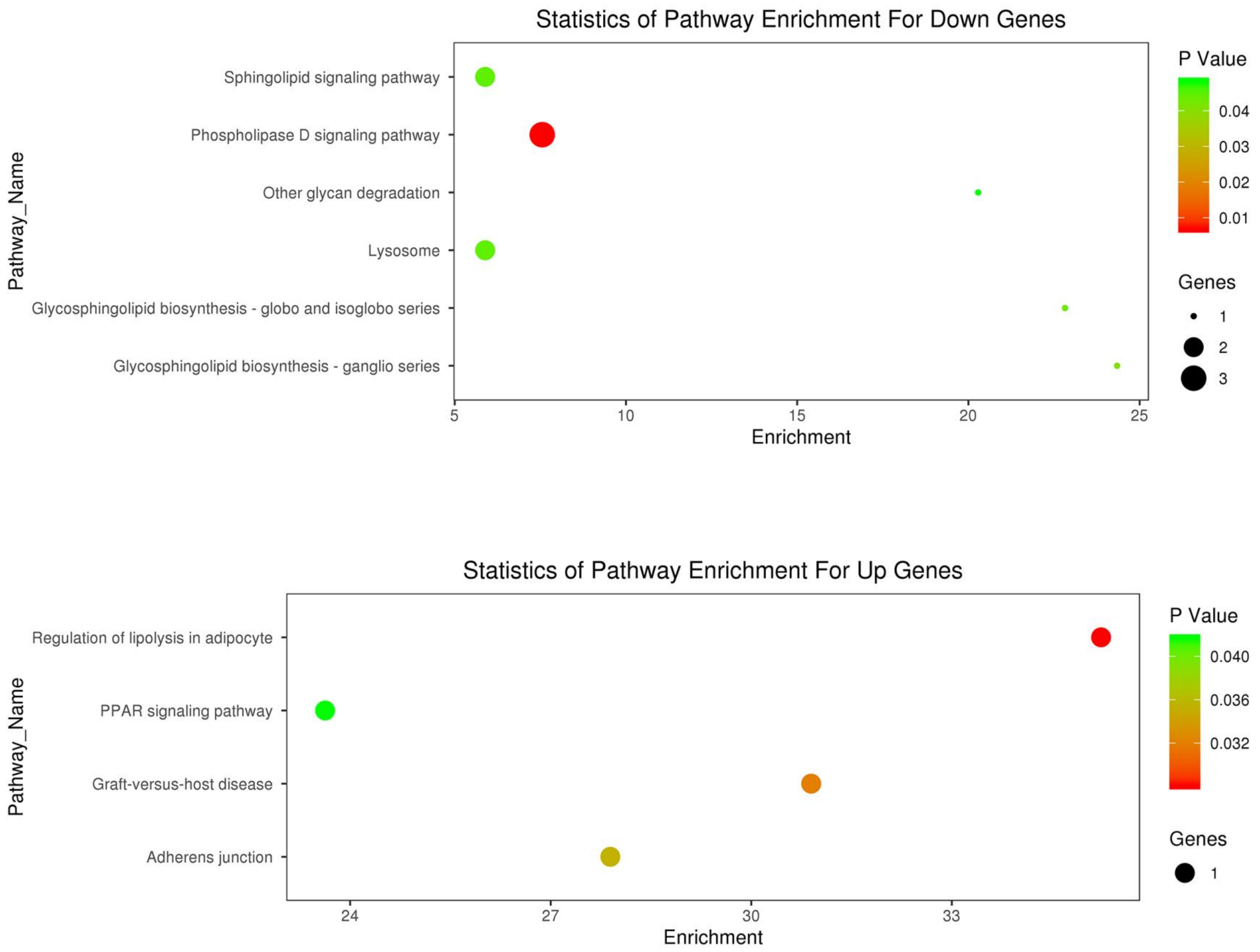

Supplementary Figure 25. Annotation of KEGG pathways selectively altered in *Rbx1*-deficient Treg cells

Top panel: enriched pathways from down-regulated genes;

Bottom panel: enriched pathways from up-regulated genes.

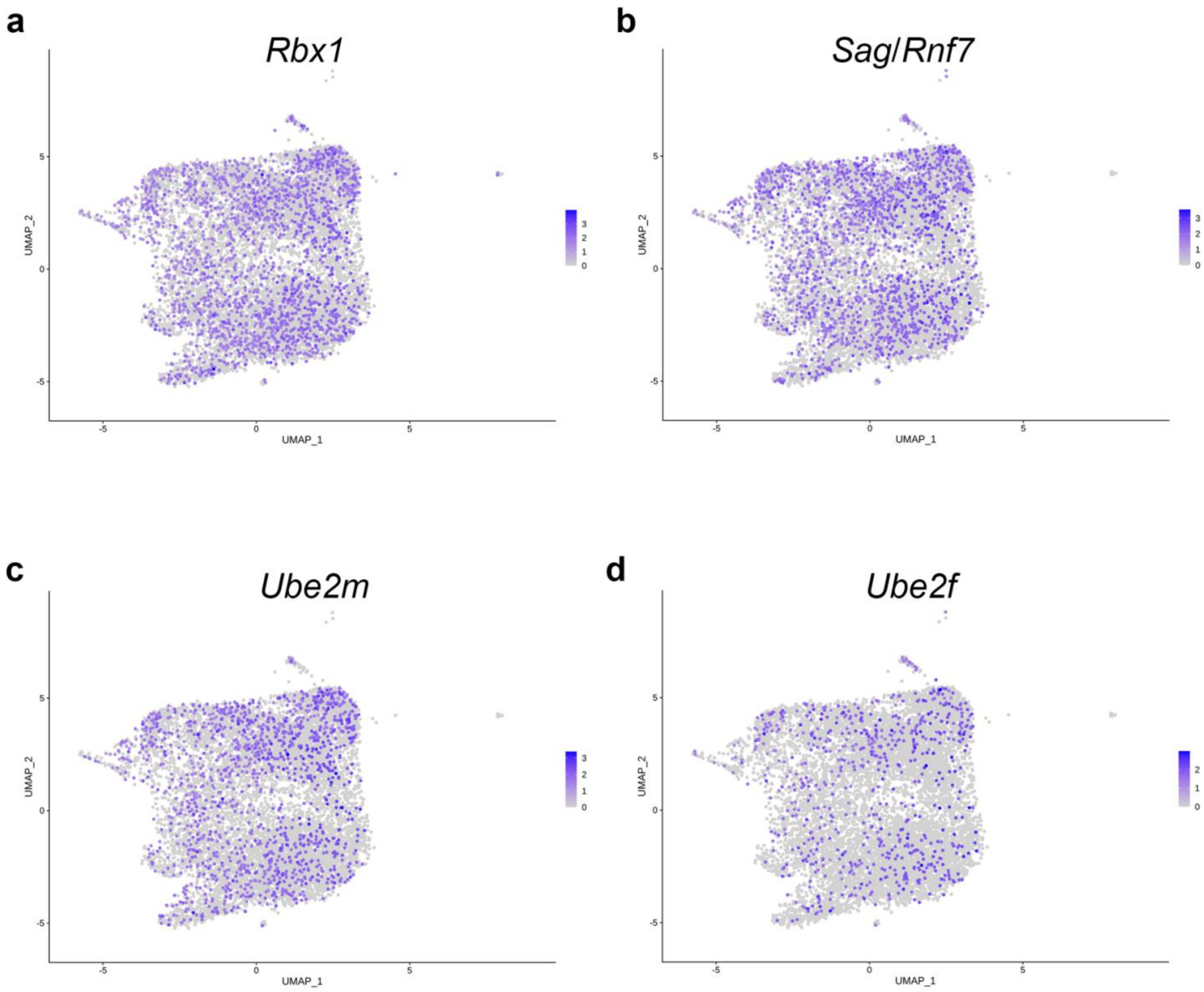

Supplementary Figure 26. The expression of neddylation E2 and E3 encoding genes in Treg cells revealed by sc-RNA sequence

(a) Expression of *Rbx1* in Treg cells.

(b) Expression of *Sag*/*Rnf7* in Treg cells.

(c) Expression of *Ube2m* in Treg cells.

(d) Expression of *Ube2f* in Treg cells.

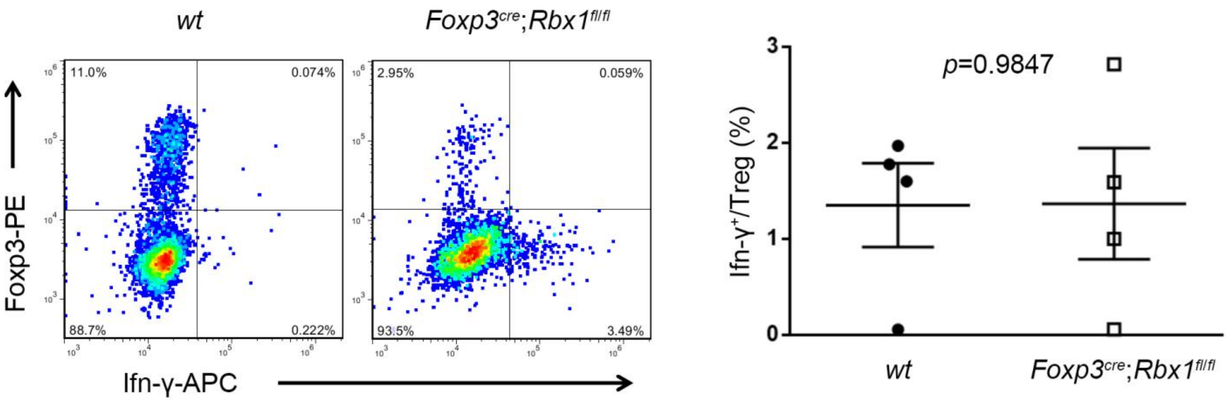

Supplementary Figure 27. Rbx1-deficient Treg cells does not produce Ifn-γ

Ifn-γ production in Treg cells (the Foxp3^{+}Ifn-γ^{+} populations in the top right-hand corner) from *wt* and *Foxp3^{cre}*;*Rbx1$^{fl/fl}$* mice (p19 for representative image; p19-23, *n* =4 biologically independent samples from both male and female mice for statistics analysis).

All error bars represent the SEM, data are presented as mean values +/- SEM. The *p* values were calculated by Mann–Whitney test. Source data are provided as a Source Data file.

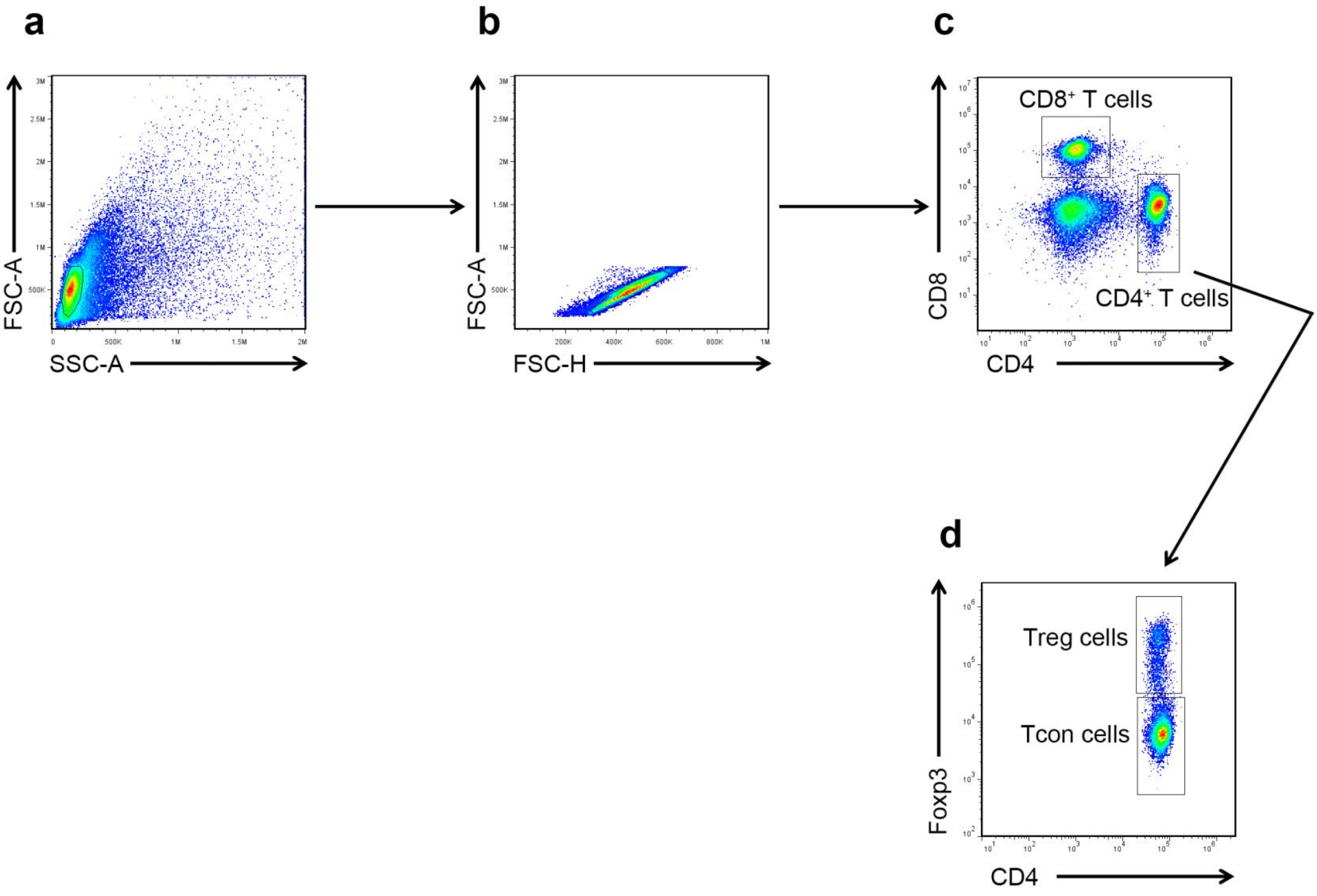

Supplementary Figure 28. Diagram of FACS gating strategy for T cells

The “Tcon cells” populations were subjected to further analyses in Fig. 1f, 2e, 2f, 2g, 6e, 7e, 8e, 8f, S1b, S1c, S16b, S16c and S17 as indicated respectively.

The “Treg cells” population were subjected to further analyses in Fig. 3d, 3e, 6a, 8b, 8c, S7c, S7d, S15b and S15g as indicated respectively.

The strategy of Fig. S28d was used in Fig. 2b, 6g, 8a, S1d, S7a and 16d respectively.

The “CD8^{+} T cells” population were subjected to further analyses in Fig. S4a and S19a as indicated respectively.

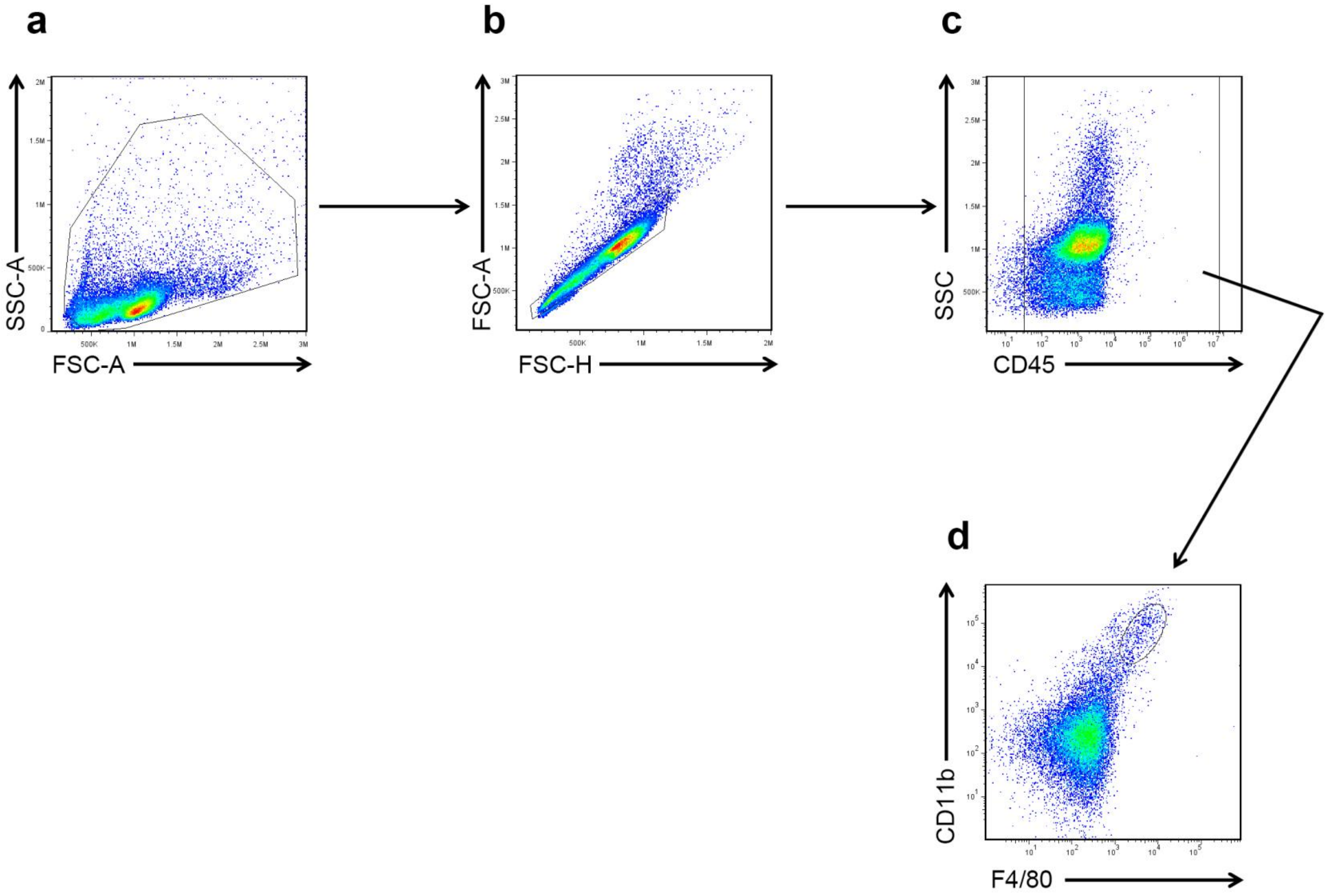

Supplementary Figure 29. Diagram of FACS gating strategy for "Fig S4b and S19b"

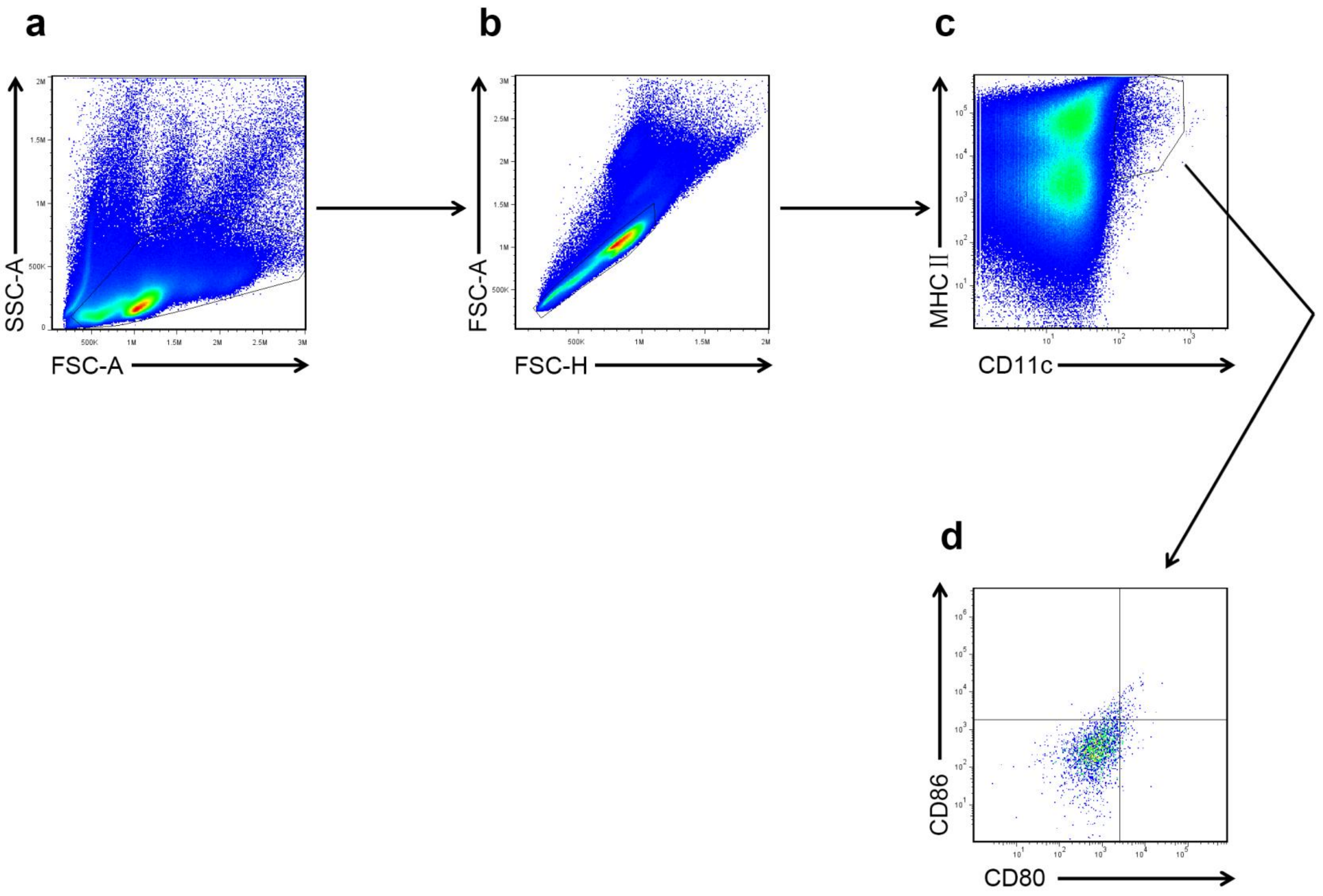

Supplementary Figure 30. Diagram of FACS gating strategy for "Fig S4c and S19c"

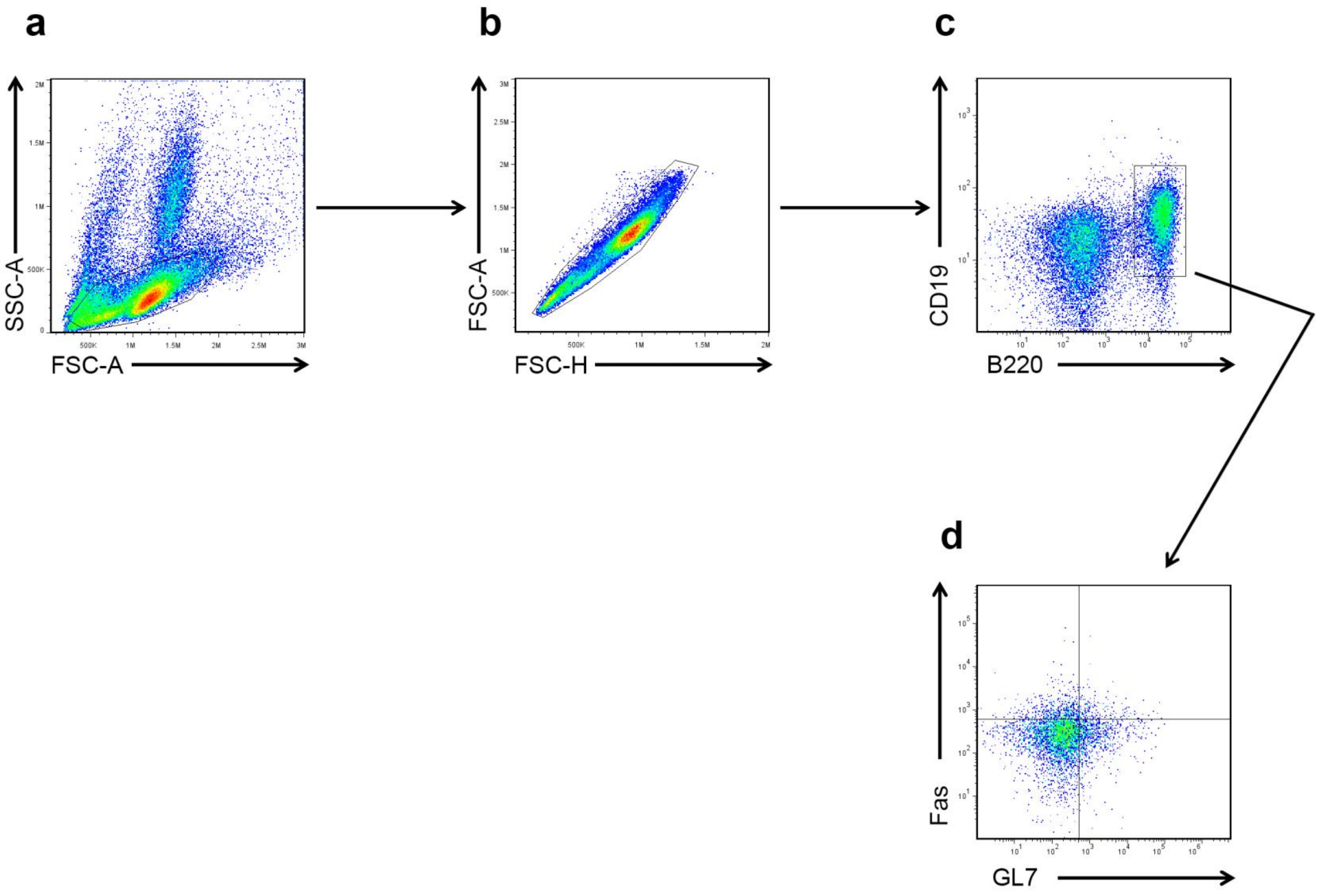

Supplementary Figure 31. Diagram of FACS gating strategy for "Fig S4d and S19d"

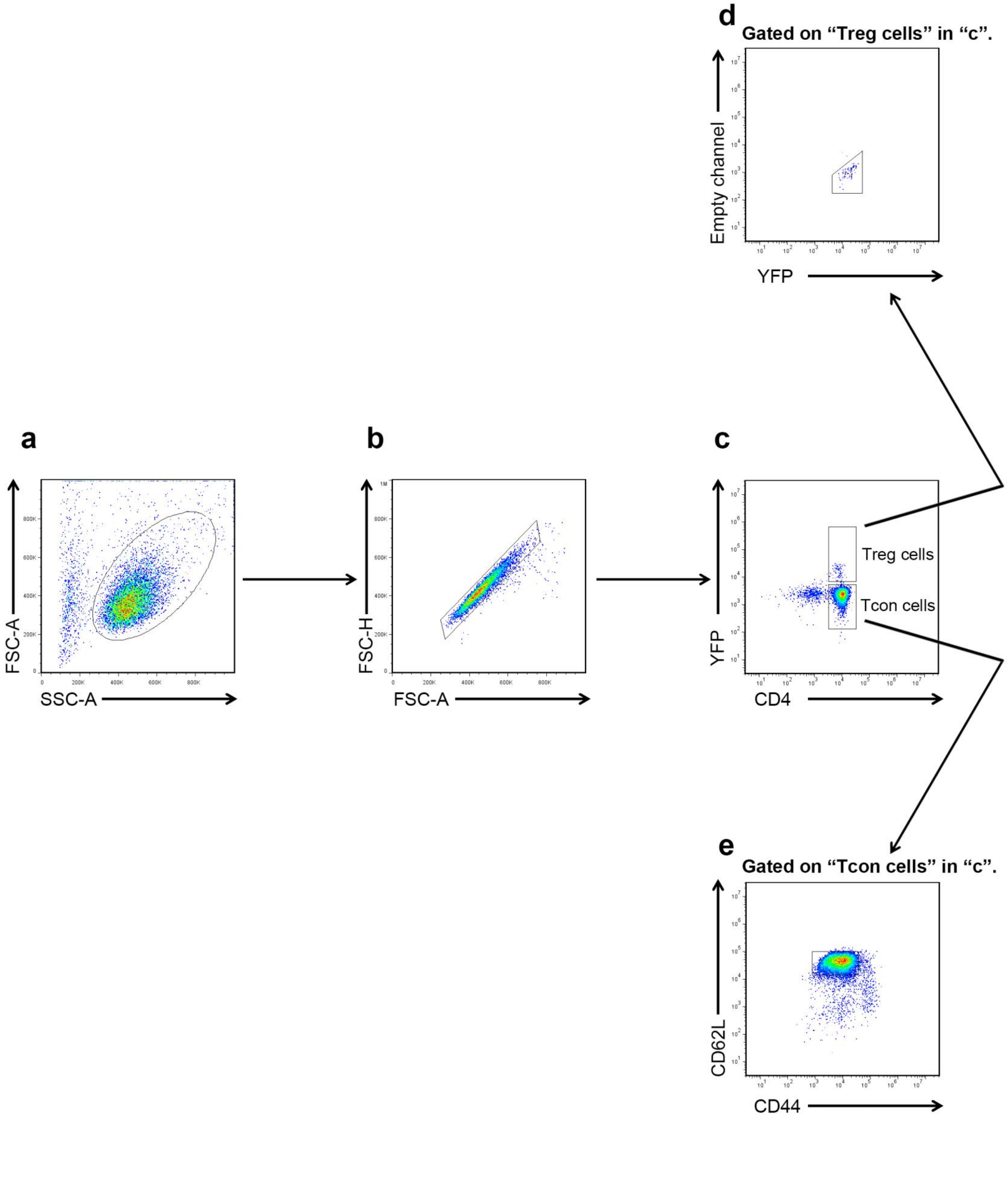

Supplementary Figure 32. Diagram of gating strategy for FACS sorting

"a, b, c, d" for $CD4^+YFP^+$ Treg cells, and "a, b, c, e" for $CD44^{lo}CD62L^{hi}$ Tnai cells.
